# mTOR and S6K1 drive polycystic kidney by the control of Afadin-dependent oriented cell division

**DOI:** 10.1038/s41467-020-16978-z

**Published:** 2020-06-24

**Authors:** Martina Bonucci, Nicolas Kuperwasser, Serena Barbe, Vonda Koka, Delphine de Villeneuve, Chi Zhang, Nishit Srivastava, Xiaoying Jia, Matthew P. Stokes, Frank Bienaimé, Virginie Verkarre, Jean Baptiste Lopez, Fanny Jaulin, Marco Pontoglio, Fabiola Terzi, Benedicte Delaval, Matthieu Piel, Mario Pende

**Affiliations:** 1 Institut Necker-Enfants Malades, 14 rue Maria Helena Vieira Da Silva, CS 61431 Paris, France; 2 Inserm, U1151, Paris, F-75014 France; 3 Université Paris Descartes, Sorbonne Paris Cité, Paris, France; 4 Institut Curie, PSL Research University, CNRS, UMR 144, F-75005 Paris, France; 5 Cell Signaling Technology INC, 3 Trask Lane, Danvers, MA 01923 USA; 6 Université de Paris, PARCC, INSERM, Equipe Labellisée par la Ligue contre le Cancer, F-75015 Paris, France; 7 Assistance Publique-Hôpitaux de Paris (AP-HP centre), Hôpital Européen Georges Pompidou, Département d’anatomo-pathologie, F-75015 Paris, France; 8 INSERM U-981, Gustave Roussy, Villejuif, France; 9 CRBM, CNRS, Univ. Montpellier, Centrosome, cilia and pathologies Lab, 1919 Route de Mende, 34293 Montpellier, France

**Keywords:** Cell division, Cell signalling, Mechanisms of disease

## Abstract

mTOR activation is essential and sufficient to cause polycystic kidneys in Tuberous Sclerosis Complex (TSC) and other genetic disorders. In disease models, a sharp increase of proliferation and cyst formation correlates with a dramatic loss of oriented cell division (OCD). We find that OCD distortion is intrinsically due to S6 kinase 1 (S6K1) activation. The concomitant loss of S6K1 in *Tsc1*-mutant mice restores OCD but does not decrease hyperproliferation, leading to non-cystic harmonious hyper growth of kidneys. Mass spectrometry-based phosphoproteomics for S6K1 substrates revealed Afadin, a known component of cell-cell junctions required to couple intercellular adhesions and cortical cues to spindle orientation. Afadin is directly phosphorylated by S6K1 and abnormally decorates the apical surface of *Tsc1*-mutant cells with E-cadherin and α-catenin. Our data reveal that S6K1 hyperactivity alters centrosome positioning in mitotic cells, affecting oriented cell division and promoting kidney cysts in conditions of mTOR hyperactivity.

## Abstract (web summary)

mTOR activation is known to generate polycystic kidneys, which show both increased proliferation and loss of oriented cell division (OCD). Here, Bonucci et al. show that loss of OCD is linked to S6K1 activation through its direct target Afadin and is separable from hyperproliferation.

## Introduction

Mammalian target of rapamycin (mTOR) is a serine/threonine kinase present in every eukaryotic cell. Its function is to integrate nutritional signals and promote growth^1^. mTOR generally stimulates macromolecule biosynthesis. At the cellular level, mTOR increases cell size, proliferation, and survival, while suppressing the autophagic degradation of intracellular material. mTOR is found in two distinct protein complexes (mTORC1 and mTORC2) with different cellular localization, nutrient sensitivity, and substrate specificity. Increasing evidence demonstrates that the constitutive activation of mTOR underlies the overgrowth phenotype of many inherited and sporadic genetic diseases. In kidneys, the great majority of genetic diseases leading to polycystosis converge in the activation of mTORC1. These include autosomal dominant polycystic kidney disease (ADPKD), type I oral-facial-digital syndrome, Tuberous Sclerosis Complex (TSC), and Birt-Hogg-Dubé syndrome (BHD)^2–5^. The requirement of mTORC1 activity for cyst formation is demonstrated by the broad efficacy of the allosteric mTORC1 inhibitor rapamycin in mouse models of these diseases^2,3,6,7^. An intense remodeling of the tissue underlies polycystic kidney development^8^. Increased proliferation rates are accompanied by alterations in cell survival, fluid secretion, dedifferentiation, alterations in basement membrane, and epithelial cell polarity. However, how the mTORC1 pathway promotes the various cellular responses in the context of polycystic kidneys remains largely unknown.

Among the genetic diseases up-regulating mTORC1, the Tuberous Sclerosis Complex (TSC) is the best characterized at the molecular level. TSC results from inherited or de novo mutations in the *TSC1* and *TSC2* genes^9^. The *TSC1* and *TSC2* gene products associate in a complex with GTPase-activating protein (GAP) activity towards the Ras homolog enriched in brain (Rheb) protein^10^. As a consequence of *TSC1/TSC2* loss-of-function mutations, the GTP-loaded form of Rheb constitutively activates mTORC1 at lysosomal membranes. TSC patients suffer from hamartomas, benign tumors in multiple organs, including the brain and kidney^9^. In addition, TSC patients display an increased risk of developing polycystic kidney disease. Extensive proteomics and biochemical studies have revealed an increasing list of mTORC1 substrates^11–13^; however, in the pathological setting of TSC, the molecular targets of mTORC1 that mediate cyst formation are unknown.

Genetic epistasis experiments in the fruit fly *Drosophila* were the first to assess the contributions of TOR and S6 Kinase (S6K) in the overgrowth of *Tsc* mutants^14^. The size of Tsc1- or Tsc2-mutant ommatidia are double that of wild type. Deletion of *Tor* causes a dramatic atrophy in both wild-type and *Tsc*-mutant background, while *S6k* deletion has a mild effect on wild-type flies, but it is sufficient to blunt *Tsc*-mutant overgrowth, precisely to the level of wild-type cells. These data suggest two important considerations: *Tor* deletion affects multiple targets involved in growth control, causing severe cellular atrophy; and the overgrowth phenotype of TSC mutants seems exquisitely sensitive to S6K inhibition, which may represent a valuable strategy against TSC-related overgrowth. Mammalian cells express two S6K homologs, S6K1 and S6K2^15,16^. They belong to the AGC family of serine/threonine kinases and may share redundant targets with Akt1-3, 90 KDa Ribosomal Protein S6 Kinase 1–4 (Rsk1-4), Serum/Glucocorticoid Regulated Kinase 1–3 (SGK1-3), and protein kinases C (PKCs)^17^. mTORC1 specifically activates S6K1 and S6K2 by phosphorylation, whereas Akt, SGK, and PKC are phosphorylated by mTORC2^18^. Since *TSC* mutations selectively up-regulate mTORC1^10^, S6Ks are the only AGC kinases activated in this disease, with the other kinases being unaffected or suppressed as a consequence of the negative feed-back regulation of mTORC1 on mTORC2^19^. S6Ks are also very sensitive to mTORC1 inhibition by rapamycin^13^. Taken together, these evidences prompted the investigation of the role of S6K in TSC pathological lesions and in rapamycin-sensitive responses.

Here we take advantage of a well-characterized model of *Tsc1* deficiency in kidney tubular cells, leading to polycystic kidneys in adult mice (*Ksp-Cre; Tsc1*^*fl/fl*^)^20^. We show that mTORC1 controls two distinct pathways in kidney epithelia, an S6K1-independent pathway regulating the rate of cell proliferation, and an S6K1-dependent pathway regulating the orientation of cell division. *S6k1* deletion in the *Tsc1*-mutant background re-establishes the correct axis of cell division and rescues the cystic phenotype. Mechanistically, we show that the S6K1 phosphoproteome is enriched in proteins involved in the cell adhesion system and actomyosin cortex. One S6K1 substrate, Afadin, is a component of cell adhesion systems and contributes to the regulation of oriented cell division.

## Results

### Loss of S6k1 rescues cyst formation without affecting proliferation

**Fig. 1:**
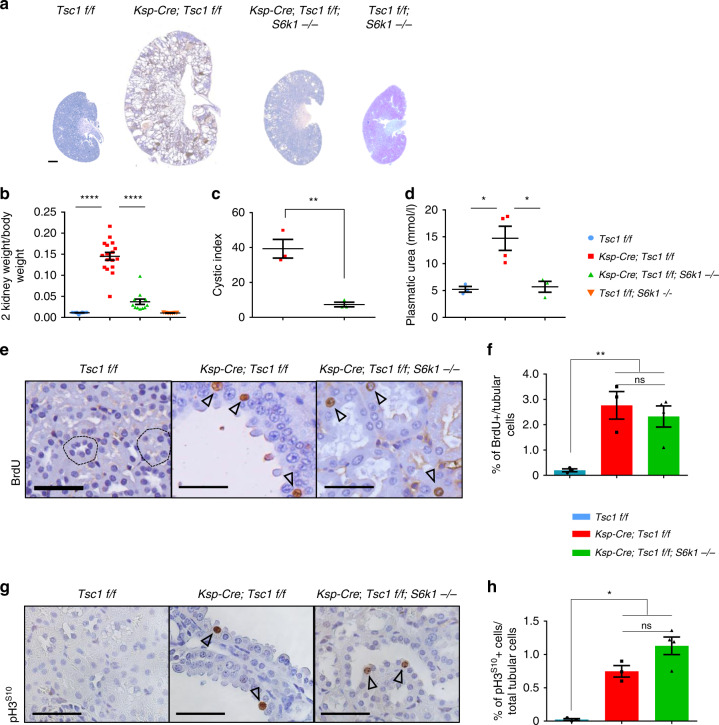
S6K1 deletion protects from cyst formation in a mouse model of renal TSC without affecting proliferation. **a** Macroscopic photos of BrdU staining at post-natal day 90 of the different genotypes indicated. Scale bar, 1 mm. **b** Kidney/body weight ratio of the indicated phenotypes at post-natal day 90. Mean  ± SEM. *n* = 12 Tsc1 f/f, *n* = 17 Ksp-Cre; Tsc1 f/f and *n* = 12 Ksp-Cre; Tsc1 f/f; S6k1 −/− mice were quantified. **c** Cystic index – (total cystic area/total kidney area) × 100 – of mice of indicated genotypes. Mean ± SEM. *n* = 3 mice/group. **d** Plasmatic urea concentrations (mmol/l) were determined in the indicated genotypes at post-natal day 20. Mean ± SEM. *n* = 3 Tsc1 f/f, *n* = 4 Ksp-Cre; Tsc1 f/f and *n* = 3 Ksp-Cre; Tsc1 f/f; S6k1 −/− mice were quantified. **e** Representative pictures and **f** relative quantifications of BrdU staining at post-natal day 90 of the indicated genotypes. The percentage of BrdU-positive cells on the total number of tubular cells was counted in 10 different fields of each section. Dotted circles show transversal cuts of normal tubules. Empty arrows point at positive cells. Mean ± SEM. *n* = 3 Tsc1 f/f, *n* = 3 Ksp-Cre; Tsc1 f/f and *n* = 4 Ksp-Cre; Tsc1 f/f; S6k1 −/− mice were quantified *n* = 3 mice/group. Scale bar, 20 µm. **g** Representative pictures and **h** relative quantifications of pH3^S10^ staining at post-natal day 90 of the different genotypes indicated. The percentage of pH3^S10^-positive cells on the total number of tubular cells was counted in 10 different fields of each section. Empty arrows point at positive cells. Mean ± SEM. *n* = 3 Tsc1 f/f, *n* = 3 Ksp-Cre; Tsc1 f/f and *n* = 4 Ksp-Cre; Tsc1 f/f; S6k1 −/− mice were quantified. Scale bar, 20 µm. Source data are provided as a Source Data file.

To score for the epistatic interaction of *Tsc1* and *S6k1* in polycystic kidney development, we compared *Ksp-Cre; Tsc1*^*fl/fl*^ with *Ksp-Cre; Tsc1*^*fl/fl*^*; S6k1*^−/−^ kidneys. *Ksp-Cre* expression drives recombination of floxed alleles in kidney tubular cells starting from E14.5^21^. Using a confetti reporter, recombination was detected in both the cortex and medulla (Supplementary Fig. 1a, b). As previously reported^20,22,23^, *Tsc1* deletion resulted in kidney overgrowth and cyst formation (Fig. 1a and Supplementary Fig. 2a, b). At postnatal day 90 (P90), the kidney to body weight ratio was 14-fold greater than wild type (Fig. 1b). Strikingly, kidney overgrowth of *Tsc1* mutants was blunted by the deletion of *S6k1*, and the cystic index was also sharply reduced (Fig. 1c). This led to improvements in kidney tissue and function as assessed by histology (Supplementary Fig. 2b) and plasmatic urea levels (Fig. 1d), respectively. Since increased cell proliferation might contribute to cyst formation, cell cycle progression was scored by anti-Bromodeoxyuridine (BrdU) staining, after a 2-h-pulse with BrdU, and anti-phospho Histone H3 staining. The former labels S-phase cells and the latter mitotic cells. As shown in Fig. 1e–h, *Tsc1* deletion caused a more than 20-fold increase in tubular cell proliferation. Surprisingly, *S6k1* inactivation did not affect the proliferation rate of *Tsc1*-mutant cells. Taken together, our data indicate that it is possible to separate cell proliferation and cyst formation driven by mTORC1. In the absence of S6K1, mTORC1 hyperactivation still drives cell proliferation, but cyst formation is strongly prevented. One expected consequence would be tubule elongation rather than enlargement in *Ksp-Cre; Tsc1*^*fl/fl*^*; S6k1*^−/−^ kidneys. Since the great majority of the distal part of the nephron resides in the medulla, a lengthening of the tubular system often results in a decrease of the cortex to medulla thickness ratio. Consistently, in *Ksp-Cre; Tsc1*^*fl/fl*^*; S6k1*^−/−^ kidneys, the cortex to medulla ratio was significantly reduced as compared to wild type, demonstrating a lengthening of the tubular system (Supplementary Fig. 3).

The fact that the proliferation observed in *Tsc1*-deficient kidneys does not depend on S6K1 was confirmed in another mouse line of *Tsc1* deficiency, *CAGGCre-ER*^*TM*^*; Tsc1*^*fl/fl*^. As we previously reported^24^, this ubiquitously expressed and temporally regulated Cre transgenic allele allows the mosaic deletion of *Tsc1* in all tissues after tamoxifen (TM) administration, recapitulating the multisystemic features of the disease. In kidneys, the overgrowth phenotype of *CAGGCre-ER*^*TM*^*; Tsc1*^*fl/fl*^ mice was milder as compared to *Ksp-Cre; Tsc1*^*fl/fl*^ mice, leading to a 9-fold increase in kidney to body weight ratio at P90 (Supplementary Fig. 4a). Of note, *S6k1* deletion was sufficient to partially blunt the overgrowth, while the combined deletion of *S6k1* and *S6k2* did not further reduce kidney weight. Consistent with the *Ksp-Cre; Tsc1*^*fl/fl*^ model, *S6k1* deficiency did not have an impact on tubular cell proliferation, but rather on cyst formation (Supplementary Fig. 4b, c). Thus, S6K1 activity is required for robust cyst formation in mouse models of TSC.

### mTORC1/S6K1 activation and cell size alterations precede cyst formation

**Fig. 2:**
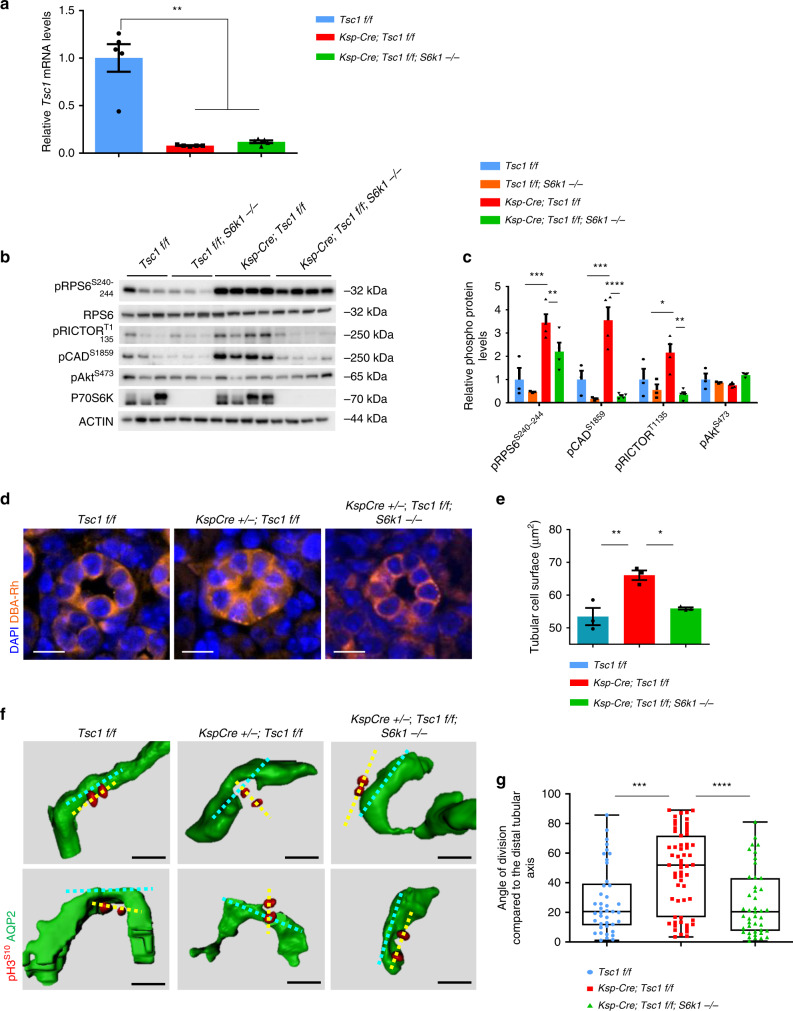
S6K1 hyperactivation is responsible for the aberrant orientation of cell division in TSC renal disease. **a** RT–QPCR for *Tsc1* expression from mouse kidneys of indicated genotypes. Data are normalized to the control mice. Mean ± SEM, *n* = 5 mice/group. **b** Western blots of mouse whole-kidney extracts at pre-cystic age (post-natal day 20) of the indicated genotypes. Mice are randomly fed. Antibodies used for the immunoblot analysis are indicated. Actin was used as a loading control. **c** Densitometry analysis and fold change over control of the indicated phospho-proteins relative to actin. Mean ± SEM. *n* = 3 Tsc1 f/f, *n* = 3 Tsc1 f/f; S6k1 −/−, *n* = 4 Ksp-Cre; Tsc1 f/f and *n* = 4 Ksp-Cre; Tsc1 f/f; S6k1 −/− mice were quantified. **d** Representative pictures and **e** relative measurements of tubular cell surface area at post-natal day 10 of the indicated genotypes. Roughly *n* = 50 tubular sections from five different fields of each section were measured. Mean ± SEM. *n* = 3 mice/group. Scale bar, 10 µm. **f** Representative 3D reconstructed images of mitotic divisions (anaphase) of renal tubular cells, longitudinally oriented in *Tsc1*^*fl/fl*^ and *Ksp-Cre; Tsc1*^*fl/fl*^*; S6k1*^*−/−*^ mice and aberrantly oriented in the *Ksp-Cre; Tsc1*^*fl/fl*^ mice. Mice were killed at precystic (post-natal day 20) stage. The tubular axis orientation is marked with a cyan dotted line, the mitosis orientation with a yellow dotted line. **g** Quantification of the mitotic division orientation at post-natal day 20 in of Tsc1 f/f (*n* = 41), Ksp-Cre; Tsc1 f/f (*n* = 8059) and Ksp-Cre; Tsc1 f/f; S6k1 −/− (*n* = 49 = 43) distal tubules, in four mice per genotype. To assess the significance of the data, a Mann–Whitney *U* test was used. Median ± interquartile range and Min and Max values. *n* = 5 mice/group. Source data are provided as a Source Data file.

*Ksp-Cre*-mediated recombination led to similar losses of Tsc1 expression in *Tsc1*^*fl/fl*^ and *Tsc1*^*fl/fl*^*; S6k1*^−/−^ kidneys, as assessed by *Tsc1* mRNA expression (Fig. 2a). In *Tsc1*-mutant kidneys, tubular enlargement was not observed in the first 3 weeks of postnatal life; however, at precystic stage P20, S6K activity was already up-regulated in *Ksp-Cre; Tsc1*^*fl/fl*^ kidneys (Fig. 2b). S6K1 deletion impaired phosphorylation of Carbamoyl-Phosphate Synthetase 2, Aspartate Transcarbamylase, And Dihydroorotase (CAD) and Rapamycin-insensitive companion of mTOR (RICTOR), known to be S6K1-specific substrates (Fig. 2c)^25,26^. The phosphorylation of ribosomal protein S6 (RPS6) was not completely inhibited in S6K1-deficient kidneys, owing to the presence of S6K2^15^. Consistent with the TSC1/2 complex selectively controlling mTORC1^19^, Akt phosphorylation by mTORC2 was not increased in TSC mutants. These changes in mTORC1/S6K1 signal transduction correlated with S6K1-dependent changes in tubular cell size at precystic stage (Fig. 2d, e), a reliable read-out of S6K1 activity^27,28^. Thus, mTORC1/S6K1 activation and cell size alterations preceded cyst formation in mouse TSC kidneys.

### Loss of S6k1 rescues mis-oriented cell division in a TSC background

The control of cell division orientation was previously proposed as a causative factor concurring to polycystic kidney formation^29–32^, a concept that has been challenged more recently^33,34^. We therefore asked whether *Tsc1* deletion altered the orientation of cell division in an S6K1-dependent manner. A tissue clearing method was set-up to measure in 3D the angle of cell division relative to the tubular lumen axis at P20. After tissue clearing, mitotic cells were labeled using phospho-histone H3, and the tubular lumen was decorated using anti-aquaporin2 (AQP2) antibodies, which label collecting ducts and distal tubules, or wheat germ agglutinin (WGA), which mainly labels proximal tubules. Mitotic events were analyzed exclusively in epithelial cells lining the tubules and not in underlying stromal cells (Supplementary Fig. 5a). In wild-type sections, the axis of cell division tended to be parallel to the longitudinal axis of the tubular lumen, as the angle of cell division relative to the lumen was <20° in the great majority of mitotic events (Fig. 2f, g and Supplementary Fig. 5b). However, in *Tsc1* mutants, the angle of cell division was random, with an even distribution from 0° to 90° in both proximal and distal tubules. S6K1 inactivation in TSC mutants re-established the correct distribution of cell division angles. In conclusion, S6K1-dependent misorientation of cell division is an early, possibly causative, event concurring to cyst formation in a context of proliferative TSC-mutant kidneys. It is likely that non-cell autonomous effects could be a major reason of tubule distortion, including humoral factors, disturbed urinary flow, or altered cellular adhesion between mutant cells and neighboring wild-type cells. It has been reported that Tsc1 deletion can also lead to non-cell autonomous activation of the mTORC1 pathway in epithelial cells^23^. Therefore, mTORC1 is likely to alter OCD in both Tsc1-mutant and neighboring wild-type cells, consistent with the general efficacy of rapamycin to blunt polycystic disease.

**Fig. 3:**
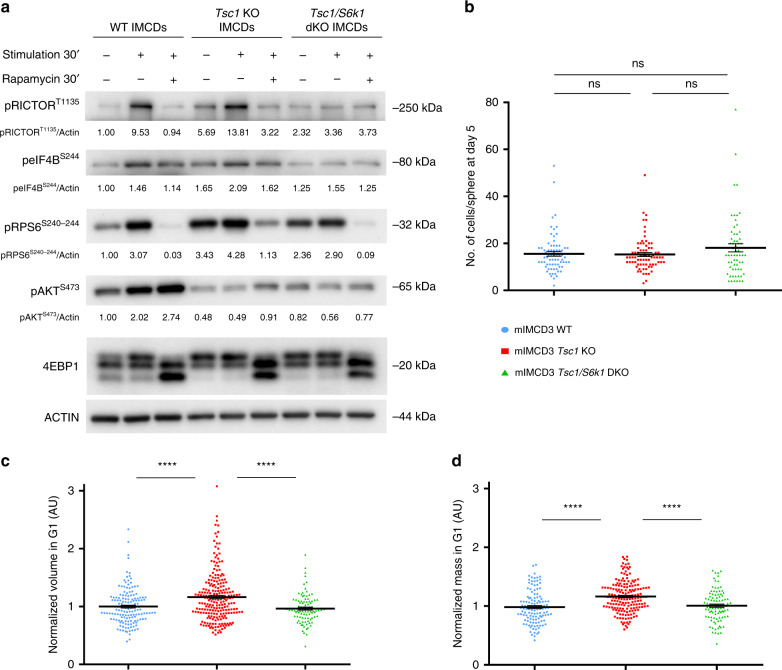
Characterization of CRISPR/Cas9-mediated deletion of *TSC1* and *S6K1* in mIMCD3 cells. **a** Cells were serum starved overnight and subsequently amino acid starved with EBSS (Earle’s Balanced Salt Solution) for 1.5 h. They were then either collected in starvation or stimulated with complete medium for 0.5 h and collected or treated with 20 nM rapamycin in complete medium for 0.5 h and then collected. Cell lysates were probed with the indicated antibodies to characterize the effects of the loss of *Tsc1* and *Tsc1/S6k1* on mTORC1 signaling pathways in the cell lines. Densitometry analysis and relative quantification of the phosphorylated forms of the indicated proteins are reported under each blot. Actin was used as a loading control. **b** Cells were trypsinized, plated as single cells in Matrigel and cultured for 5 days before being fixed with 4% PFA and stained with DAPI to quantify the number of cells per sphere derived from a single cell. An average of 100 spheres pooled from three independent experiments were quantified for each genotype. Horizontal lines represent the mean ± SEM. **c** Volume and **d** mass were analyzed by optical measurement at G1 (2.5 h after mitotic division) for the different genotypes. An average of 100 cells pooled from three independent experiments were quantified for each genotype. Horizontal lines represent the mean ± SEM. Source data are provided as a Source Data file.

To dissect the cellular and molecular alterations underlying misoriented cell division by hyperactive mTORC1/S6K1 signaling, mouse inner medullary collecting duct-3 (mIMCD3) cells were edited using CRISPR-Cas9 to delete the *Tsc1* gene alone or in combination with *S6k1* (Supplementary Fig. 6a). As expected, *Tsc1* deletion conferred constitutive activation of the mTORC1 pathway even in serum- and amino-acids-free conditions, as shown by the band-shift of the mTORC1 substrate 4E-BP1 and the phosphorylation of the S6K1 substrates Rictor, rpS6 and Eukaryotic Translation Initiation Factor 4B (eIF4B) (Fig. 3a). In addition, mTORC1 activation suppressed mTORC2-dependent Akt phosphorylation, due to the negative feed-back loop of mTORC1/S6K1 on mTORC2. Thus, the different branches of mTOR signaling are properly regulated by the TSC1 and S6K1 transduction elements in these edited kidney cell lines.

mIMCD3 cells can be maintained in culture for many passages due to SV40 transformation and are commonly used to study kidney epithelial organization, as they form polarized spheres after 5 days in Matrigel-embedded cultures from a single cell. Cell numbers were comparable among the genotypes at the end of the 5-day-spheroid assays (Fig. 3b). Cell volume and mass were measured in single cells by fluorescent dye exclusion^35^. *Tsc1*-deficient cells in G1 phase displayed an S6K1-dependent 20% increase in both cell volume and cell mass (Fig. 3c, d). Accordingly, cell density, defined as the ratio of mass to volume, did not differ. (Supplementary Fig. 6b). The differences in cell volume among the three genotypes were also observed at the beginning of mitosis and during mitotic round-up (Supplementary Fig. 6c). Volume heterogeneity of the two daughter cells was not observed, as the ratio of the larger to smaller daughter cell was equivalent (Supplementary Fig. S6d). Taken together, while *Tsc1*-deficient mIMCD3 cells do not have a proliferative advantage over wild type in an SV40-transformed background, they display an S6K1-dependent increase of volume and mass throughout the cell cycle, consistent with cell size measurements in kidneys (Fig. 2d).

**Fig. 4:**
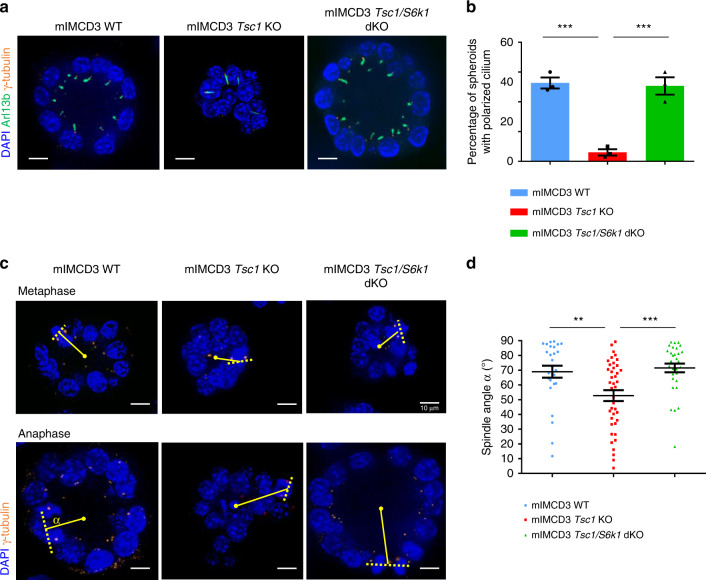
Hyperactive S6K1 affects spindle orientation in sphere assays in vitro. **a** Representative images of 5-day mIMCD3s spheres immunolabeled with γ-Tubulin, Arl13b, and DAPI to quantify spheres with polarized centrosomes and primary cilia. Only spheres with a visible lumen and with all the centrosomes and primary cilia correctly localized on the luminal (apical) side were considered as normal. Scale bar, 10 µm. **b** Quantification of the percentage of spheres with polarized primary cilia on total number of spheres per genotype. The ratio was calculated on *n* = 70 spheres per experiment. The histograms are an average of three independent experiments. Mean ± SEM. **c** Representative images of 5-day mIMCD3s spheres immunolabeled with γ-tubulin and DAPI to measure spindle orientation. Yellow dotted lines indicate the spindle vectors, solid lines indicate the radius. Scale bar, 10 µm. **d** Quantification of spindle angles relative to the apical-basal axis in 5-day spheres. Each dot represents a mitotic event, three independent experiments were analyzed. Horizontal lines represent the mean ± SEM. Source data are provided as a Source Data file.

The sphere formation assay in 3D-Matrigel embedded cultures is a common method to evaluate oriented cell division and cell polarity^36^. Wild-type cells embedded in Matrigel formed polarized spheroids, with interphase cells displaying a γ-Tubulin-positive centrosome at the apical surface and an Arl13b-positive primary cilium pointing toward the lumen (Fig. 4a). More than 50% of wild-type spheres had polarized cilia after 5 days (Fig. 4b). In contrast, *Tsc1*-deficient cells formed spheres with mispositioned primary cilia and small lumens. To quantify the reduction of lumen dimension, we calculated the ratio between the lumen diameter and number of surrounding cells, which showed a significant reduction (Supplementary Fig. 7a, b). Strikingly, *S6k1* deletion was sufficient to rescue polarized sphere formation and lumen size.

To confirm if defects in cell division orientation were also observed, we monitored mitotic spindle orientation, since it determines the axis of cell division^37^. In 3D renal cultures, the centrosome duplicates at the end of interphase, at the apical membrane. During mitosis, the two centrosomes relocate perpendicularly to the radius of the spheroid to the lateral sides of the cell to form and orient the mitotic spindle, returning to the apical surface after cytokinesis. To assess mitotic spindle orientation, centrosomes were labeled with γ-Tubulin antibodies and the mitotic phases were recognized by DAPI staining of DNA (Fig. 4c). To calculate the axis of cell division, the centroid of the spheroid in the middle z-plane and the midpoint of the spindle axis were determined. By using apical markers, 3D reconstitution and orthogonal projections, it was possible to unambiguously define the centroid of the spheroid, including the *Tsc1* mutants (Supplementary Fig. 7c). Next, the angle between the spindle axis and the center-to- was measured. In the majority of metaphase and anaphase wild-type cells, this angle was close to 90° (Fig. 4d). However, In *Tsc1-*mutant cells, the spindle axis was misoriented. This defect was rescued by the deletion of *S6k1*, suggesting a causal link between S6K1 hyperactivity and spindle pole mispositioning.

**Fig. 5:**
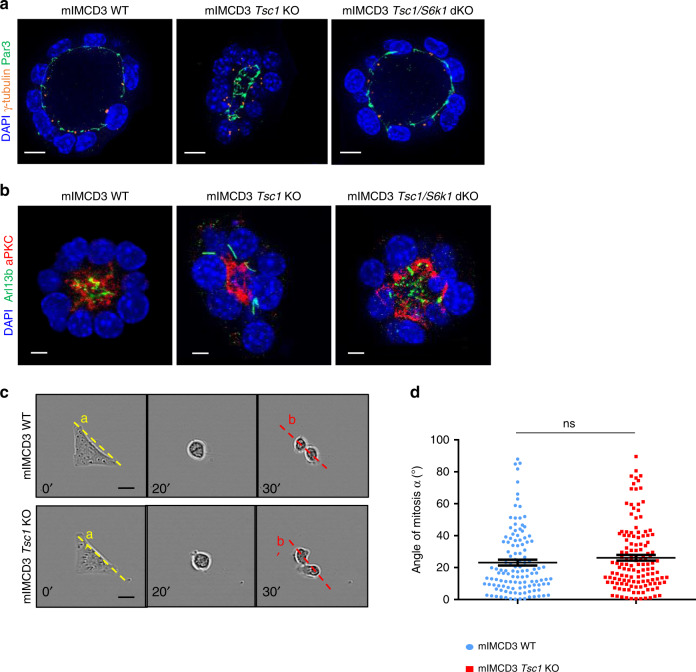
Misoriented cell division and centrosome position in *Tsc1* mutant cells is not due to intrinsic defects. **a** Representative images of 5-day mIMCD3s spheres immunolabelled with γ-tubulin, Par3 and DAPI to show the localization of the centrosomes compared to the apical markers. Scale bar, 10 µm. **b** Representative images of 5-day mIMCD3s spheres immunolabelled with Arl13b, aPKC, and DAPI to show the localization of the primary cilia compared to the apical markers. Scale bar, 5 µm. **c** Representative images of cells cultured on L shape patterns and recorded by time-lapse microscopy for the different genotypes. Scale bar, 20 µm. **d** Distribution of mitotic spindle angles relative to pattern orientation (α^b^−α^a^) at anaphase onset. An average of 120 angles from three independent experiments were quantified for each genotype. Horizontal lines represent the mean ± SEM. Source data are provided as a Source Data file.

Spindle pole position in mitosis is tightly regulated by cell geometry and influenced by the following factors: (i) determinants of the apical complex (aPKC and Par3/Par6); (ii) shape and contractility of the actomyosin cortex; (iii) the NuMA/LGN complex which exerts pulling forces for astral microtubules at the cell cortex; (iv) the interaction with the basal matrix through the integrin system; (v) cell-to-cell junctions through the cadherin system^37^. As a first attempt to dissect the contributions of these factors in the TSC phenotype, the primary cilia were stained relative to the apical domain detected by Par3 and aPKC antibodies (Fig. 5a, b). Additional apical markers, such as zona occludens-1 (ZO-1) and Ezrin, confirmed that *Tsc1*-mutant cells were able to properly locate the apical complex at the luminal side (Supplementary Fig. S8a, b), ruling out the possibility that a defect in the establishment of apical polarity contributes to spindle misorientation. In addition, the localization of the basal marker Integrin beta 1 (ITGB1) did not differ among the genotypes (Supplementary Fig. S8c).

Then we asked whether the defect in *Tsc1* mutants was cell autonomous or depended on cell–cell interactions, single cells were grown on fibronectin-coated L-shape micropatterns. In this system, cells rely on the integrin system to define the basolateral domain. If there are no defects in the integrin system and no intrinsic defects in the actomyosin cortex and mitotic spindle, cells will divide along their longer axis (Fig. 5c)^38,39^. Strikingly, *Tsc1* mutants correctly divided similarly to the wild-type cells (Fig. 5d). Taken together, these data point to a rather non-cell autonomous defect in *TSC1*-mutants to correctly position the spindle, depending on the presence of neighboring cells. In fact, in Matrigel embedded cultures or in vivo, cells coordinate their division according to tissue polarity, orienting the spindle by communication through cell–cell junctions^40^.

### Phoshoproteomic screening identifies Afadin as a S6k1 substrate

**Fig. 6:**
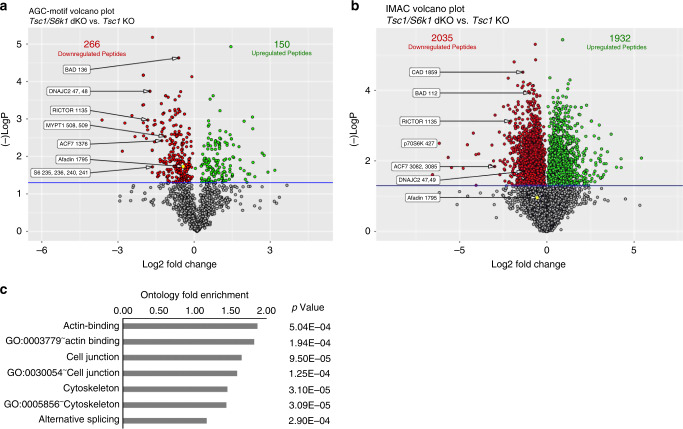
Phosphoproteomic screening of *Tsc1* single knockout versus *Tsc1/S6k1* double knockout cells. **a** Volcano plots of AGC kinase-motif antibody enrichment and **b** IMAC enrichment. The *x*-axis shows log2-ratio for phosphopeptides between samples and the *y*-axis shows the −log10 *p*-value. The horizontal blue bar represents the statistical cut-off of significance (*p* value of 0.05). Red dots represent downregulated phospho-peptides in the Tsc1/S6k1 double knock-out genotype and green dots represent up-regulated phospho-peptides. Afadin phospho-peptide is represented in yellow. The numbers in the chart represent the number of statistically significant differentially enriched peptides. Representative peptides and their sites of modification are shown. **c** Gene ontology analysis, using DAVID, showing statistically significant gene set enrichments comparing the IMAC statistically significant differentially phosphorylated proteins versus the entire IMAC enrichment set. Source data are provided as a Source Data file.

The published literature on S6K substrates does not readily suggest putative targets involved in mitotic spindle positioning and intercellular adhesion cues. Thus, we exploited a phosphopeptide enrichment method coupled with liquid chromatography tandem mass spectrometry (LC-MS/MS)^41^ to uncover the S6K1-dependent phosphoproteome by comparing *Tsc1-* and *Tsc1/S6k1*-deficient mIMCD3 cells. The cell lines were cultured in serum- and amino acids-free conditions, which accentuated the sensitivity of *Tsc1-loss* for mTORC1 signaling (Fig. 3a). To have a broad representation of phosphopeptides, two different methods of phosphopeptide enrichment were performed in parallel. The first method took advantage of commercially available antibodies recognizing phosphorylated serine/threonine (S/T) preceded by arginine (R) residues, a motif commonly recognized by AGC kinases including S6K. We combined antibodies recognizing the RXXS/T (where X indicates any amino acid) and RXRXXS/T motifs, as their patterns of phosphoprotein detection in western blots were not superimposable (Supplementary Fig. 9). The second method used Fe-NTA Immobilized Metal Affinity Chromatography (IMAC) to enrich for phosphopeptides. In total, 15,444 unique phosphopeptides were identified after IMAC enrichment and 1355 after AGC-motif enrichment, with a common subset of 839 phosphopeptides, indicating that the two methods are complementary, allowing broader coverage of the phosphoproteome (Supplementary Table 1). The great majority of known S6K substrates were identified and quantified in the analysis (Fig. 6a, b, Supplementary Table 2). Out of 27 S6K substrates reported in the literature in different cell types, 12 were significantly down-regulated in *Tsc1/S6k1* mutant cells as compared to *Tsc1* mutants from the IMAC screen and 9 from the AGC screen. The combination of the two methods covered more than 50% of reported S6K substrates, highlighting the ability to find proteins t of interest using this methodology.

Next, we performed gene ontology (GO) analyses to evaluate whether the differentially expressed phosphopeptides were enriched in specific functional classes over the total phosphopeptides detected in each screen. The AGC-motif screen did not show any significant classes due to the limited set of detected peptides. However, the IMAC screen revealed that GO terms for actin binding, cell junctions, cytoskeleton, and alternative splicing were significantly overrepresented in the differentially expressed phosphopeptides (Supplementary Table 3). To select putative direct substrates of S6K1, we focused on phosphopeptides in both screens that were down-regulated in *Tsc1/S6k1*-deficient cells and contained an AGC-motif (Fig. 6a, b, Supplementary Table 4). Among the proteins with a function in the regulation of oriented cell division were the following: Afadin, an actin binding and adapter protein regulating the nectin and cadherin adhesion systems^42^; slingshot protein phosphatase 1 (SSH1), phosphatase for the actin depolymerase cofilin^43^; microtubule actin crosslinking factor 1 (Macf1), spectraplakin binding actin and microtubules^44^; phosphatidylinositol 4-Kinase α (PI4K), regulating endocytic trafficking^45^; myosin phosphatase-targeting subunit 1 and 2 (MyPT1 and MyPT2), myosin phosphatases regulating actomyosin contractility^46^; missing in metastasis protein (Mtss1), cortactin-interacting protein^47^.

**Fig. 7:**
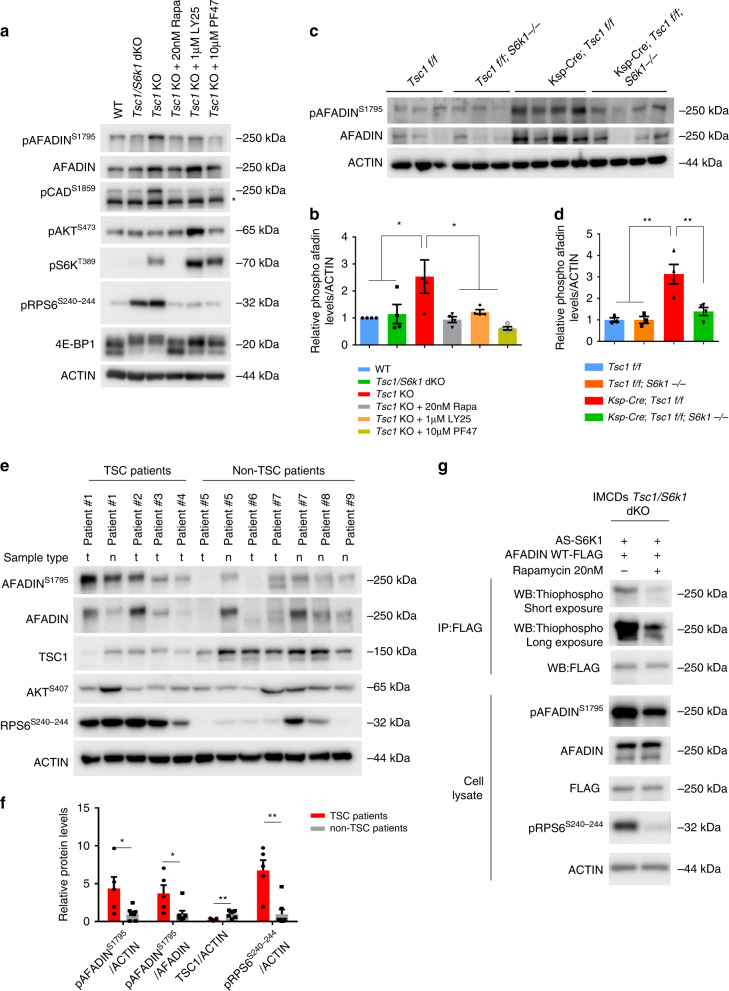
Afadin is phosphorylated by S6K1 in *Tsc1*-mutant conditions. **a** Cells were serum starved overnight and subsequently amino acid starved with EBSS (Earle’s Balanced Salt Solution) for 1.5 h with or without the indicated treatments. They were then collected and cell lysates were probed with the indicated antibodies. Asterisk represents non-specific cross-reactive bands. **b** Densitometry analysis and relative quantification of the phosphorylated forms of the indicated proteins. Actin was used as a loading control. Mean ± SEM. *n* = 4 independent experiments were analyzed. **c** Western blots of mouse whole-kidney extracts at pre-cystic age (post-natal day 20), randomly fed, and probed with the indicated antibodies. **d** Densitometry analysis and relative quantification of the phosphorylated form of Afadin. Actin was used as a loading control. Mean ± SEM. *n* = 3 Tsc1 f/f, *n* = 3 Tsc1 f/f; S6k1 −/−, *n* = 4 Ksp-Cre; Tsc1 f/f, and *n* = 4 Ksp-Cre; Tsc1 f/f; S6k1 −/− mice were analyzed. **e** Western blot of protein lysates extracted from patient samples. Renal tumors (t) or normal tissue adjacent to renal tumor (n) were collected from TSC patients and non-TSC patients. Protein lysates then probed with the indicated antibodies. **f** Densitometry analysis and fold change of TSC patients over non-TSC patients of the indicated proteins relative to the indicated loading control. Mean ± SEM. *n* = 5 TSC patients, *n* = 7 non-TSC patients were analyzed. **g** Analog-sensitive S6K1 was co-transfected in *Tsc1/S6k1* dKO mIMCD3 cells with FLAG-Afadin-WT. An in vivo kinase assay was performed in the presence of 6‐Bn‐ATP‐γ‐S both in presence and in absence of 20 nM rapamycin. After immunoprecipitation using an anti-FLAG antibody, the thio-phosphorylation of Afadin was revealed by western blot using an anti‐thiophosphate ester antibody. Expression levels of Afadin, phospho-S1795-Afadin, FLAG and phospho-RPS6 were revealed by western blot on total extracts using the indicated antibody. Source data are provided as a Source Data file.

Afadin emerged as a prime candidate for validation, as loss-of-function mutants in a kidney tubular cell line displayed defects in sphere formation and oriented cell division^48^. Moreover, Afadin is a known component of cell–cell junctions, potentially coupling intercellular adhesions and cortical cues to spindle orientation. Antibodies recognizing the S6K1-dependent phospho-S1795 site of Afadin are commercially available, allowing site-specific validation of the LC-MS/MS data. Phospho-Afadin levels were increased in *Tsc1*-deficient mIMCD3 cells, while the levels in *Tsc1/S6k*1-deficient cells were comparable to wild type (Fig. 7a, b). In addition, pharmacological inhibition of mTORC1 by rapamycin and of S6K1 by LY-2779964 and PF-4708671 decreased Afadin phosphorylation in *Tsc1*-deficient cells after a 2-h treatment. The regulation of Afadin phosphorylation by Tsc1 and S6k1 was also confirmed in knock-out mouse kidney extracts (Fig. 7c, d). Next, Afadin phosphorylation was assessed in human kidney surgical samples from TSC patients and non-TSC patients (Fig. 7e). Whenever possible, tumor samples (t) were compared to adjacent normal tissue (n). The kidney tumors from TSC patients were hybrid oncocytic-chromophobe renal cell carcinomas (patients #1 and 2) or angiomyolipomas (patients #3 and 4). The non-TSC patient samples were cystic nephromas (patient #5), oncocytomas (patient #6), clear cell renal carcinomas (patients #7 and 8), or well-differentiated liposarcomas (patient #10). Of note, Afadin phosphorylation was significantly increased in TSC patients, suggesting a clinical relevance of the findings in cellular and mouse models.

To demonstrate whether S6K1 could directly phosphorylate Afadin, *Tsc1/S6k1*-deficient cells were transfected with a point-mutant of S6K1 conferring sensitivity to bulky ATP-analogs^49^. The analog-sensitive S6K1 was able to thio-phosphorylate Afadin in a rapamycin-sensitive manner (Fig. 7g), demonstrating that Afadin is a direct S6K1 substrate. Since we observed that total Afadin levels correlated with the extent of phosphorylation (Fig. 7a, c), we asked whether its phosphorylation state affected protein stability, as previously proposed^50^. Cycloheximide treatment suggested that the half-life of Afadin was longer in *Tsc1-* versus *Tsc1/S6k1*-deficient cells, correlating with phosphorylation status (Supplementary Fig. 10a, b). In addition, mRNA levels of Afadin correlated with the activation of the mTORC1/S6K1 pathway (Supplementary Fig. 10c), indicating the control of Afadin expression at different levels, precluding a clear-cut determination of the effects on protein stability.

**Fig. 8:**
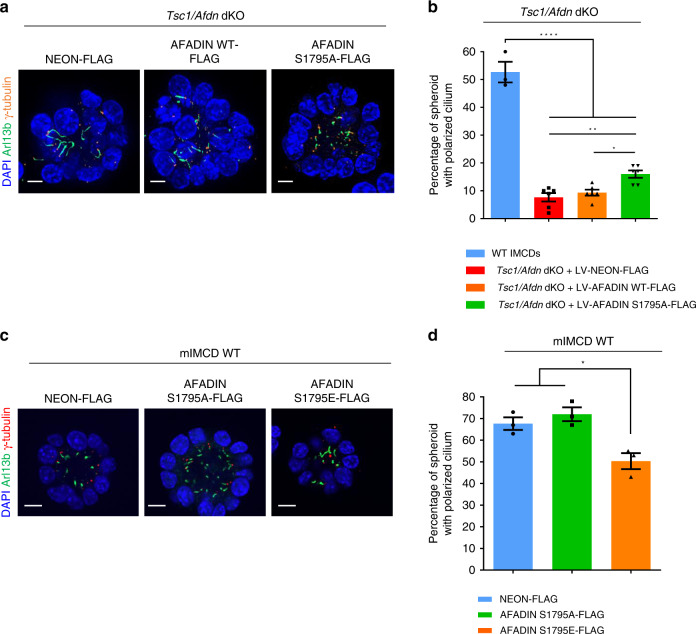
Effects of Afadin phospho-mutants on sphere formation. **a** Representative images of 5-day spheres from cells *Tsc1/Afdn* dKO transduced with lentivirus FLAG-Neon, FLAG-Afadin-WT, or FLAG-Afadin-S1795A. After fixation, spheres were immunolabeled with γ-Tubulin, Arl13b, and DAPI to quantify spheres with polarized centrosomes and primary cilia. Scale bar, 10 µm. **b** Quantification of the percentage of spheres with polarized primary cilia on total number of spheres from cells WT and *Tsc1/Afdn* dKO transduced with lentivirus FLAG-Neon, FLAG-Afadin-WT, or FLAG-Afadin-S1795A. The ratio was calculated on *n* = 70 spheres per experiment per each genotype. The histograms are an average of six independent experiments (for WT *n* = 3 independent experiments). Mean ± SEM. **c** Representative images of 5-day spheres from WT mIMCD cells transduced with lentivirus FLAG-Neon, FLAG-Afadin-S1795A (phospho-deficient) or FLAG-Afadin-S1795E (acidic phospho-mimetic). After fixation, spheres were immunolabeled with γ-Tubulin, Arl13b and DAPI to quantify spheres with polarized centrosomes and primary cilia. Scale bar, 10 µm. **d** Quantification of the percentage of spheres with polarized primary cilia on total number of spheres per genotype from WT mIMCD cells transduced with lentivirus FLAG-Neon, FLAG-Afadin-S1795A, or FLAG-Afadin-S1795E. The ratio was calculated on *n* = 70 spheres per experiment per each genotype. The histograms are an average of three independent experiments. Mean ± SEM. Source data are provided as a Source Data file.

To investigate its functional role, *Afadin* expression was disrupted using CRISPR-Cas9 in both wild-type and *Tsc1*-deficient mIMCD3 cells, followed by lentiviral transduction of wild-type (wt) Afadin, serine-to-alanine-1795 phospho-deficient mutant (S1795A) or vector control (Supplementary Fig. 11a). Both Afadin constructs were expressed similarly, below endogenous levels, and correctly localized at the membrane (Supplementary Fig. 11b). *Afadin* knock-out and *Afadin/Tsc1* double knock-out cells had impaired formation of polarized spheres (Fig. 8a, b, and Supplementary Fig. 11c). Re-expression of WT-Afadin and S1795A-Afadin induced comparable rescue of sphere formation in *Afadin* knock-out cells (Supplementary Fig. 11c), likely because in the WT background, Afadin is not hyperphosphorylated. However, in the *Tsc1/Afadin* double knock-out cells, only the S1795A-Afadin phospho-mutant could partially rescue the ability to form polarized spheres (Fig. 8a, b). This demonstrates that aberrant Afadin phosphorylation by S6K1 hyperactivation in *Tsc1* mutants is partly responsible for the OCD defect. In addition, the serine-to-glutamate-1795 phosphomimetic (S1795E) mutant was introduced in wild-type cells to ask whether this mutation was sufficient to alter sphere formation. Of note, both the phospho-deficient (S1795A) and the phospho-mimetic (S1795E) mutants were expressed at comparable levels (Supplementary Fig. 11b), but the phospho-mimetic led to disturbances in cellular adhesion, as indicated by formation of thinner and less regular Afadin-junctions (Supplementary Fig. 11f, g). Moreover, the ability to form spheres with polarized cilium was reduced in cells expressing S1795E-Afadin (Fig. 8c, d). These data suggest that Afadin phosphorylation impairs the adhesion system leading to defects in oriented cell division.

**Fig. 9:**
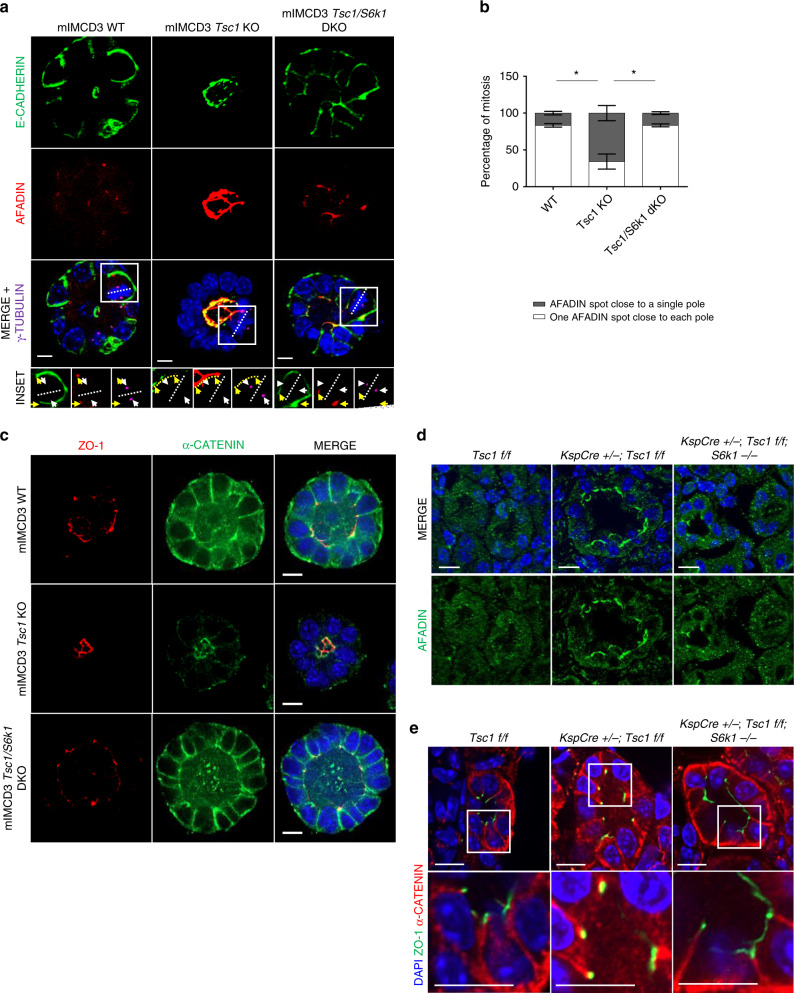
*Tsc1*-deficient spheres display altered Afadin/Cadherin-based adhesion system localization. **a** Representative images of 5-day mIMCD3s spheres of the indicated genotypes immunolabelled with Afadin (red), E-cadherin (green), γ-Tubulin (magenta) antibodies, and DAPI to show the relative localization of Afadin and E-cadherin in interphase and relative to the mitotic spindle poles in 3D spheroids. Insets show higher magnification of one mitotic cell per genotype, to show the relative localization of Afadin and E-cadherin in mitotic cells. In the insets, yellow arrows indicate the localization of the Afadin spots. White arrows indicate localization of the poles of the mitotic spindles. A dotted yellow line indicates Afadin staining in the whole apical domain of mitotic cells. Scale bar, 10 µm. **b** Percentage of mitosis showing one Afadin spot adjacent to each spindle pole (white), or perturbed afadin localization adjacent to a single spindle pole (dark gray). An average of 10 mitosis were analyzed per experiment per each genotype. The histograms are an average of three independent experiments. Mean ± SEM. **c** Representative images of 5-day mIMCD3s spheres of the indicated genotypes immunolabelled with α-catenin, ZO-1 and DAPI to show the loss of baso-lateral presence of α-catenin in the Tsc1 KO, with a concomitant appearance of an apical mislocalization relative to the apico-lateral marker ZO-1. Scale bar, 10 µm. **d** Representative pictures of pre-cystic post-natal day 20 mice, immunolabelled with Afadin antibody to show the localization of the protein in the indicated genotypes. Scale bar, 10 µm. **e** Representative pictures of pre-cystic post-natal day 20 mice, immunolabelled with α-catenin, ZO-1 and DAPI to show the localization of the proteins in the indicated genotypes. Source data are provided as a Source Data file.

Therefore, Afadin localization was determined in mitotic cells during sphere formation. E-Cadherin-based adhesions are the main found in tubular cells. As Afadin is involved in their regulation^51^, the relative localization of the two proteins was assessed by immunofluorescence. γ-Tubulin was used to determine the spindle poles in mitotic cells. In wild-type cells, Afadin was mainly at adherens junctions (AJ) in the two apicolateral domains of both mitotic and interphase cells (Fig. 9a and insets). The tight-junction protein ZO-1 was also localized at the apicolateral domains (Supplementary Fig. 12a). In *Tsc1*-mutant cells, Afadin staining was more abundant and diffuse throughout the whole apical domain (Fig. 9a and Supplementary Fig. 12a). During mitosis of wild type and *Tsc1/S6k1*-mutant cells, the two Afadin spots at the AJs were found at opposite sides of the spindle equator, each one close to one spindle pole (Fig. 9a, b). However, in the majority of *Tsc1* mutant cells, the Afadin spots were on the same side of the spindle equator, close to a single pole, and in other mitotic events, it was impossible to distinguish two spots, but rather a localization throughout the whole apical domain. E-Cadherin was mainly basolateral in wild type and *Tsc1/S6k1*-mutant cells during interphase and mitosis (Fig. 9a). Strikingly, in *Tsc1*-mutant cells, E-Cadherin was aberrantly found at the apico-lateral domain (Fig. 9a and Supplementary Fig. 12b, c). Additional components of the Cadherin adhesion system, including α-catenin, β-catenin, and the polarity protein Scribble, also displayed apical localization instead of baso-lateral in *Tsc1* mutants (Fig. 9c and Supplementary Figs. 12b and 13a, b). Importantly, Afadin and α-catenin also decorated the apical surface in *Tsc1*-mutant kidneys at pre-cystic stage (Fig. 9d, e). These data suggest that mTOR/S6K1 hyperactivity alters spindle orientation relative to Afadin-positive AJs and this correlates with a modification of the cadherin-based adhesion system.

## Discussion

Polycystic kidney is a common pathological consequence of hyperactive mTORC1, as observed in TSC and additional inherited diseases^2–5^. Here we identify S6K1 as a direct mTORC1 substrate required for cyst formation. S6K1 deletion affects the orientation but not the rate of cell division in TSC mutant mice, thus disconnecting the need for these two cell cycle alterations at the origin of polycystic kidneys. Centrosome mispositioning in mitotic *Tsc1*-mutant cells accompanies the defect in oriented cell division, as observed in Matrigel-embedded cultures, where cells rely on cell-junctions to position the centrosome. Performing an unbiased and comprehensive S6K1-phosphoproteome in the TSC background, we highlight that peptides whose phosphorylation is S6K1-dependent, are mainly regulators of intercellular junctions and actomyosin cortex. Among them, Afadin is a direct S6K1 substrate involved in the control of oriented cell division, possibly by coupling intercellular adhesions and cortical cues to the mitotic spindle.

An increasing list of mTORC1 targets has been implicated in the physiological responses of macromolecule biosynthesis and autophagy control. However, the molecular effectors of aberrant mTORC1 signaling remain uncharacterized. Here we address a pathway affecting OCD, by showing in vivo and in 3D cultures the involvement of S6K1. Interfering with other major outputs of mTORC1 activity, such as autophagy and cap-dependent translation, does not seem to affect cyst formation. Autophagy mutants and 4E-BP1/4E-BP2-deficient mice do not recapitulate a polycystic kidney phenotype^52,53^. We can speculate that the S6K1-dependent phosphoproteome orchestrates a pleiotropic response required for the intense tissue remodeling in polycystic kidneys. S6K1 is known to regulate proteins involved in protein and nucleotide synthesis, folding, RNA splicing, and insulin sensitivity. Interestingly, our MS data also suggest the existence of a class of S6K1 substrates with a role in intercellular adhesion and regulation of the actomyosin cortex. Taking advantage of Afadin phospho-specific antibodies and mutant cells, we demonstrated that Afadin is a direct S6K1 substrate participating in OCD control.

Afadin is a 200-kDa adapter protein that stabilizes adherens junctions (AJs), by interacting with both the nectin and E-cadherin adhesion systems^42^. The Afadin PDZ domain directly binds and crosslinks the intracellular domain of Nectin to the actomyosin cortex. In addition, Afadin strengthens the association between p120-catenin and E-cadherin, thus inhibiting E-cadherin endocytosis^51^. The localization of Afadin at the cellular cortex also plays an important role during cell division. In epithelial cells, its accumulation at the AJs provides a cue for the orientation of the spindle poles at the lateral cortex during mitosis. Mechanistically, Afadin can interact with LGN, which in complex with NuMA recruits the motor protein dynein to pull the astral microtubules^54^. Consistently, Afadin-deficient renal tubular cells have misoriented cell division^48^.

A previous study has revealed that in breast cancer cells, Afadin phosphorylation by Akt promotes Afadin stability and cell migration^50^. In addition, Afadin knock-down experiments have demonstrated that one of its functions is the commitment of cells to adhere or migrate^50,55^. In the present study, we show that the Afadin phosphomimetic mutant alters the cell–cell junctions while Afadin phosphorylation in TSC-mutant kidney cells is accompanied by a striking alteration of the E-cadherin adhesion system, which becomes localized at the apical surface. It is tempting to speculate that Afadin phosphorylation may somehow represent a switch between migration and adhesion, perhaps by tuning the interaction between the Nectin/Afadin-Cadherin/Catenin systems. TSC mutations have been previously shown to result in alterations of both cell–matrix and cell–cell adhesion systems. TSC1 inactivation in cell lines leads to a reduction of focal cell–matrix adhesion, while TSC1 overexpression increases focal adhesion and actin stress fibers through ezrin and Rho signaling^56^. Similarly, TSC2 overexpression activates cell attachment and reduces chemotactic cell migration^57^. Cell lines from Eker TSC2 mutant rats display intracellular E-cadherin retention in the Golgi apparatus, increased invasion and ability to grow in suspension^58–60^. However, these studies did not reveal the underlying direct mTORC1-dependent mechanism. Our data open a new perspective, indicating that the deregulation of the mTORC1-S6K1-Afadin axis may contribute to cystogenesis by altering the adhesion and spindle orientation program.

OCD has been proposed to regulate kidney tubule morphogenesis, and its disruption has been associated with development of polycystic kidney disease (PKD)^29–31,61–64^. However, more recently it has been suggested that OCD disruption may not necessarily lead to cyst formation^33,34^. Our results reconcile these apparent contrasting findings. Taking advantage of a unique system, we could separate cystogenesis into its two crucial components. Our results show that the inactivation of *Tsc1* leads to both hyper-proliferation and loss of OCD that, together, lead to the onset of a cystic kidney phenotype. Here we demonstrate that the concomitant loss of S6K1 prevents the impairment of OCD without affecting the cell proliferation. Remarkably, in this setting, the preservation of OCD combined with hyper proliferation leads to a harmonious overgrowth of the kidney without cyst formation. Ultimately, the concept that concomitant OCD disruption and hyper-proliferation are both necessary to develop cysts is in agreement with previous studies claiming that simple OCD loss or simple hyper-proliferation are not sufficient^33,34,65^.

The great efficacy of rapamycin derivatives in several mouse models of PKD raised hope for a cure for these devastating diseases^2,3,7,66^. In human clinical trials, rapamycin derivatives blunted angiomyolipoma growth in TSC patients^67^, while the results for ADPKD were moderate or negative^68–70^. The general consensus is that the doses of rapamycin derivatives used in these trials were not adequate to completely inhibit mTORC1 activity in kidneys, while higher doses showed intolerable extrarenal side effects during long-term treatment. However, high doses and long-term treatments of rapamycin may also affect mTORC2 targets in vivo, possibly concurring to toxic effects including insulin resistance^71^. The definition of mTORC1/S6K1 action on OCD and cyst formation may suggest future therapeutic developments.

## Methods

### Mice

*Tsc1*^*fl/fl*^ and *Ksp-Cre* mice were obtained from The Jackson Laboratory. The generation and genotyping of *S6k1*-mutant and *S6k1/S6k2*-double mutant mice are described elsewhere^15^. *S6k1*-mutant mice were first crossed with *TSC1*^*fl/fl*^ mice and then with *Ksp-Cre* mice. CAGGCre-ER mice were obtained from The Jackson Laboratory and crossed with *Tsc1*^*fl/fl*^ mice as described elsewhere^24^. The R26R-Confetti mice were obtained from The Jackson Laboratory and crossed first with *Tsc1*^*fl/fl*^ mice and then with *Ksp-Cre* mice. All animal studies were approved by the Direction Départementale des Services Véterinaires (Prefecture de Police, Paris, France; authorization number 75–1313) and the ethical committee of Paris Descartes University. Mice were housed in a 12-h light/dark cycle and fed a standard chow. Control mice were Cre-negative littermates of mutant mice.

### Reagents

Pharmacological inhibitors were added to cells for the time and at the concentration, as specified. Rapamycin (#1292; Tocris) was dissolved in 100% ethanol (25 mg/ml), aliquoted and stored at −20 °C. A 20-nM working solution was prepared in fresh media before use and cells were treated for the indicated time. PF-4708671 (#S2163) and LY2584702 Tosylate (#S7704) were purchased by Selleckchem and were dissolved in 100% DMSO, aliquoted and stored at −20 °C. In all, 10 µM and 1 µM working solution were prepared in fresh media before use and cells were treated for the indicated time.

For in vivo treatment, Temsirolimus (LC Laboratories) was dissolved in 100% ethanol (25 mg/ml), aliquoted, and stored at −20 °C. 1 mg/ml working solution was prepared in 5% Tween-80, 5% PEG400, and 86% PBS before injection. Mice were injected i.p. with vehicle or temsirolimus (5 mg/kg) every other day for 2 weeks before killing.

### Antibodies

For immunoblotting: anti-Rictor (#2114), anti- phospho Rictor Thr1135 (#3806), anti-DNAJC2 (#12844), anti-phospho DNAJC2 (Ser47) (#12397), anti-CAD (#93925 S), anti- phospho CAD (Ser1859) (#12662), anti-S6K1 (#2708), anti phospho S6K1 (Thr389) (#9234), anti-phospho Akt (#9271), anti-phospho RPS6 (Ser240/244) (#2215), anti-eIF4B (#3592), anti-phospho eIF4B (Ser422) (#3591), anti-phospho 4E-BP1 (Thr37/46) (#2855), anti-Afadin (for cells) (#13531), and anti-phospho Afadin (Ser1795) (#5485) were purchased from Cell Signaling Technology; anti-TSC1 (#sc- 377386) was purchased from Santa Cruz Biotechnology, Inc; anti-β actin (#A5441), anti- α Tubulin (#T5168), and anti-FLAG M2® (1/1000; #F1804) were purchased from Sigma-Aldrich. For immunofluorescence, immunohistochemistry, and immunocytofluorescence: anti-Afadin (1/500; #A0224), anti-BrdU (1/200; #BMC9318), anti-γ-Tubulin (1/500; #T5326), anti-α-catenin (1/200; #C2081), and Phalloidin-TRITC (1/500; #P1951) were purchased from Sigma-Aldrich; anti-phospho Histone H3 (Ser10) (1/400; #ab5176) and anti-NuMA (1/200; #ab109262) were purchased from Abcam; rhodamine labeled Dolichos Biflorus Agglutinin (DBA) (1/200; #RL-1032) was purchased from Vector Laboratories; anti-Arl13b (1/400; #17711-1-AP) was purchased from Proteintech; anti-LGN (1/200; #ABT174), anti-PAR3 (1/200; #07-330), and anti-β-catenin (1/200; #06-734) were purchased from Merk Millipore; anti-aPKC (1/100; #sc-17781), anti-Scribble (1/200; #sc-28737), and anti ZO-1 (1/200; #sc-33725) from Santa Cruz Biotechnology, Inc; anti-FGFR1OP (FOP) (1/200; #H00011116-M01) was from Abnova; anti E-cadherin (1/400; # 13-1900) was purchased from Thermo Scientific; anti-Ezrin (1/200; P81) and anti-Integrin-β1 (1/200; P5D2) were purchased by DSHB.

For immunofluorescence after clearing: anti-phospho Histone H3 (Ser10) (1/200); anti AQP2-Alexa Fluor ® 488 conjugated (1/200; # sc-515770) was purchased from Santa Cruz Biotechnology, Inc; FITC-labeled Wheat germ agglutinin (WGA) (1/100; #FL-1021) was purchased from Vector Laboratories; TO-PRO™-3 Iodide (1/1000; #T3605) from Thermo Fisher Scientific.

### Cell culture

The mIMCD3 cell line was purchased from ATCC and were cultured in DMEM (#10566032; Gibco) supplemented with 10% fetal bovine serum, 20 U/ml penicillin/streptomycin (#15140122; Gibco), and 1 mM sodium pyruvate (#11360070; Gibco). For spheroid formation, the protocol used is described elsewhere^36^. Briefly, cells were trypsinized and dissociated into single cells by mechanically pipetting in trypsin and resuspending in medium. Cells were then diluted to 50,000/ml in 50% normal medium and 50% growth factor-reduced Matrigel (#356230; BD Biosciences) and plated on 8-well slides (LabTek-II #155409; Thermo Scientific) with normal medium added on top. On the following day, the medium was changed to FBS-free DMEM and left until fixation.

For the generation of *Tsc1* and *S6k1* knockout (KO) lines, sgRNA oligos were cloned into pX459 (Addgene #62988) which contains the Cas9 nuclease sequence and a puromycin resistance cassette. For the generation of the Afadin KO, sgRNA oligos were similarly cloned into a pGuide vector (containing the human U6 promoter and with a modified scaffold sequence to improve stability). mIMCD3 cells were transfected with 2 µg of plasmid using *Trans*IT-X2® (MIR 6003; Mirus Bio). Forty-eight hours later, cells were trypsinized into puromycin (1 μg/ml) selection medium for 2 days and in regular medium thereafter. The colonies were allowed to grow until it was possible to pick them with a filtered tip and were transferred individually into a well of a 96-well-plate. Clones were expanded and verified for the loss of *Tsc1*, *S6k1,* and *Afdn* by western blotting. The sgRNAs sequences used to generate the clones for this study are the following: Tsc1, 5′-TCT TTG GCC GTC TCT CGT CA-3′(PAM: TGG); S6k1, 5′-TTT GAG CTA CTT CGG GTA CT-3′ (PAM: TGG); and Afdn, 5′- CTG AAG TTG TCA TGA AAC GG-3′ (PAM: CGG).

### Cloning of constructs

The 3×-Flag tagged human Afadin (pCMV-3xAF6) construct was obtained from Dundee University. The mutation of serine 1795 to alanine was performed by overlapping PCR using the following primers: Forward (5′-CAG AGA CTG TTC gCC CAG GGC CAA GAC GTG TCC AAC AAA G-3′) and Reverse (5′-TGG GcG AAC AGT CTC TGC CGC TCT TTA AAG GTC AGG TTC TCT GG-3′) and pCMV-3xAF6 as template. The mutation of serine 1795 to glutamic acid was performed by overlapping PCR using the following primers: forward (5′-AGC GGC AGA GAC TGT TCg aaC AGG GCC AAG ACG TG-3′) and reverse (5′- CAC GTC TTG GCC CTG ttc GAA CAG TCT CTG CCG CT-3′) and pLJM1-3xAF6 as template. After digestion with DpnI, the resulting PCR reaction was transformed into competent *E. coli*. Clones were verified for the presence of the amino acid substitution by sequencing. The pLJM1-Rheb (Addgene) construct was used as the backbone to create the Flag-Neon and Flag-Afadin constructs. The pLJM1-Rheb construct was digested with Nhe1/EcoR1 and a 3×-flag mNeon cassette, amplified by PCR to contain the same restriction sites, was used to replace the Rheb insert. Amplification of the wild type and S1795A Afadin constructs was performed by PCR to include Nhe1 sites at both the 5′ and 3′ ends. The pLJM1-mNeon construct was digested with Nhe1/Xba1 and replaced with the Afadin PCR constructs. Orientation was verified by digesting.

### Lentiviral production

Lentiviruses containing Neon-FLAG, Afadin WT-FLAG, Afadin S1795A-FLAG, or Afadin S1795E-FLAG were produced by the VVTG platform (SFR Necker, France) or generated by transient transfection of the lentiviral vector (plasmid) together with packaging plasmids (pBA.Rev, pTat, pGag/pol, and pVSV-G) into HEK293T cells. The supernatant containing lentiviruses was collected, centrifuged, and filtered through 0.45 µm filter and stored in aliquots at −80 °C, or immediately used to infect recipient cells. After two rounds of infection, cells were selected in puromycin (1 µg/ml) and further passaged in culture as a bulk preparation for functional assays.

### RT-qPCR

Total RNA was extracted from cells or renal tissue using the RNAeasy Mini Kit (QIAGEN), following manufacturer’s instructions. Single‐strand cDNA was synthesized from 1 μg of total RNA with SuperScript II (Invitrogen) and 125 ng random hexamer primers. Real‐time quantitative PCR was performed on MX3005P instrument (Agilent) using a Brilliant III SYBR Green QPCR Master Mix (Agilent). Relative amounts of the indicated mRNAs were determined by means of the 2^−ΔΔC^_T_ method, normalizing with *pinin* levels. The primers used for *Tsc1* mRNA were the following: forward (5′-AGC TCC GGA CCC TCC GAG AC-3′) and reverse (5′-AGC CGC TGC TCG GAT CAC CT-3′). The primer used for *Afadin* mRNA were the following: forward (5′-ACT CCC TCT ATG AAG TGC ATG T-3′) and reverse (5′-TAA GGA CGA ATC GAC CCT CTC-3′).

### Immunoblotting

Cells and tissues were lysed in NP-40 lysis buffer (20 mM Tris pH 8.8; 138 mM NaCl; 5 mM EDTA; 2.7 mM KCl; 20 mM NaF; 5% Glycerol; 1% NP-40- IGEPAL®; #CA-630; Sigma-Aldrich) supplemented with complete protease inhibitor and phosSTOP phosphatase inhibitor cocktails (Roche). Protein extracts were resolved by SDS-PAGE, transferred to PVDF membranes, and incubated with the primary antibodies. Uncropped and unprocessed scans of the most important blots are provided in the Source Data file. Human biopsies were obtained from TSC and non-TSC patients undergoing partial or total nephrectomy. Whenever possible, normal tissue adjacent to tumoral tissue was resected and analyzed separately. This study was approved by the French national ethics committee (Comité de Protection des Personnes Number 19.05.27.61541; national identification 2018-A03147-48). Uncropped western blots are provided in the Source Data file.

### Immunohistochemistry and immunofluorescence

Mouse tissues were fixed in 4% paraformaldehyde (PFA), embedded in paraffin and sectioned at 4 μm. Sections were deparaffinized and hydrated before being incubated in citrate buffer (pH6), then boiling for 3 min and then sub-boiling for 10 min. Sections were blocked in 3% normal goat serum, 1% BSA and 0.1% Triton X-100 for 30 min-1h and then incubated with the primary antibodies (or lectins) overnight at 4 °C. Next, sections were incubated with biotinylated secondary antibodies and detected with the Vectastain Elite ABC kit (Vector) and DAB chromogen system (DAKO)

For in vivo BrdU pulsing, mice were injected i.p. with BrdU (50 mg/kg) 2 h before killing. The percentage of BrdU incorporation was determined by counting BrdU-positive nuclei on the total number of nuclei among 10 distinct fields in three different animals for each condition.

For Confetti-reporter detection and immunofluorescence of Afadin, α-catenin, and ZO-1 in mouse kidney, tissues were fixed in 4% paraformaldehyde (PFA) for 10′, followed by two washes in PBS and cryopreservation in 30% sucrose at 4° over-night. Kidneys were then embedded in OCT, frozen and cut at 10 µm in a cryostat. The sections were then placed on super frost slides and post-fixed for 104 in 4% PFA. Sections were blocked and permeabilized with 7 mg/ml porcine skin gelatin, 0.5% Triton X-100 in PBS and incubated with the primary antibodies in the same solution.

For immunocytofluorescence, cells were grown on coverslips and fixed in 4% PFA (unless otherwise stated). The fixed cells were permeabilized with 0.3% Triton in PBS; blocked with 5% normal goat serum, 3% BSA in PBS; and incubated with the primary antibodies.

Spheroids in chamber slides were washed twice with PBS containing calcium and magnesium and fixed in ice-cold 4% PFA for 30-45 min at 4 °C under agitation. Once the Matrigel was dissolved, spheres were blocked and permeabilized in 7 mg/ml gelatin (porcine skin #G1890; Sigma-Aldrich) and 0.5% Triton in PBS with calcium and magnesium at RT for 1 h and then incubated with the primary antibodies diluted in the same blocking/permeabilization buffer overnight at 4 °C. Next, spheres were incubated with Alexa Fluor conjugated secondary antibodies or Phalloidin-TRITC for 4 h at RT and then DNA was stained by DAPI within the mounting medium (Vector Labs).

Immunofluorescence slides were analyzed using an ApoTome2 microscope (Zeiss) or a Confocal TCS SP5 (Leica). ImageJ software (National Institutes of Health) or Imaris v 9.2, ImarisXT, and Bitplane AG was used for quantification.

### Clearing and picture acquisition

Tissue clearing with CUBIC reagents was performed as described^72^, with some modifications. Briefly, mice were fixed by transcardial perfusion with 4% PFA, post-fixed at 4 °C for 6 h and included in agarose to be sectioned with a vibratome (Leica) sagittally at 1 mm. The sections were then incubated in CUBIC-reagent 1 at 37 °C for 1 week, washed thoroughly in PBS and then incubated at 4 °C for 30 days in primary antibody and DNA staining described in the text diluted in PBS containing 2% Triton X100 (PBS-2T), adding again the antibody after 2 weeks. Next, sections were incubated with Alexa Fluor ® secondary antibody 488 (1/200) and DNA staining in PBS-T for 2 weeks at 4°. After staining the samples were cross-linked in PBS 4% PFA for 3 h, then washed thoroughly in PBS before being transfer in Cubic-reagent 2 and acquisition.

Sections were imaged using a Lightsheet Z.1 (Carl Zeiss) with ×20 objective.

### Measurement of the angle of mitotic division

For analysis of the tissues after clearing the spindle vector Vs and the tubular axis vector Vt were generated from the coordinates (x, y, z) measured in ImageJ of the nuclei in anaphase and of the tubular staining respectively. Then the angle between the 2 vectors was measured in Excel and plotted using Prism 6 for Windows.

The measurement of spindle angle in spheroids was performed as described previously for Caco2 cells^73,74^ with some modifications. Confocal images of entire mIMCD3’s spheroids stained with γ-Tubulin and DAPI were collected and opened with ImageJ. Next, the spindle vector Vs was generated using the 3D coordinates of γ-Tubulin staining (x, y, z) of metaphase or anaphase mitosis measured in ImageJ. The midpoint (x^m^, y^m^, z^m^) of Vs was calculated and the centroid (x^c^, y^c^, z^c^) of each spheroid in the middle z-plane of the sphere was determined using ImageJ. A radial vector Vc was generated from (x^c^, y^c^, z^c^) and (x^m^, y^m^, z^m^). The projected angle between Vs and Vc was eventually calculated using Excel.

To assess spindle orientation on micropattern, cells were plated on L-shape patterned fibronectin-coated plates (Cytoo). Live imaging was then performed using Incucyte® S3. Images were acquired at 10 min intervals for 36 h. Images were deconvolved using Incucyte software and measurements of the angle of division were then performed relative to pattern orientation at anaphase onset using ImageJ software^38^.

### Cystic index analysis

The cyst formation was quantified from sagittal sections of whole kidneys^75^. Briefly, whole kidney sections were scanned using a Nanozoomer 2.0 HT (Hamamatsu) after H&E staining; 4x images for each kidney section were selected. We used Image J to quantify the surface, expressed in pixels, and covered by cysts in each kidney. We used a cut-off of 2000 pixels as the minimum surface that a cyst should have to be included in the analysis. We next calculated the ratio between the obtained cystic area and the area of the entire kidney section and expressed it as the percentage of cysts for the given kidney.

### Tubular cell surface measurement

For surface quantification, 4-μm kidney sections were stained with rhodamine labeled DBA, and an average of 80 tubular sections were measured for each genotype on three different mice using ImageJ. Briefly, the area of external profile of the tubule and the area of the lumen were measured and epithelial surface was calculated as the difference between the two areas.

### Volume and mass live optical measurement

Volume and mass were analyzed with live imaging as previously described^76^. Briefly, cells were trypsinized, counted, and resuspended in medium supplemented with 1 mg/ml Alexa Fluor 488 Dextran (MW 10 kD; Life Technologies) at a concentration of 10^6^ cells/ml before being injected in PDMS chips coated with fibronectin with a known height of 23 µm. Finally, the chamber was immersed in medium to prevent evaporation. Acquisitions were performed on a Ti inverted (Nikon) or an Axio Observer microscope (Carl Zeiss) at 37 °C, with 5% CO2 atmosphere, using a ×20 dry objective (NA 0.5 phase), or a SFluor 20× objective (NA 0.75 without phase ring) for the mass measurements. Images were acquired using MetaMorph (Molecular Devices). Images were acquired with a CoolSNAP HQ2 camera (Photometrics) for fluorescence or a PHA SICS camera (PHA SICS) for mass measurements. Images were analyzed with a custom-made MatLab program (MathWorks).

### Phosphoproteome enrichment and mass spectrometry

For phosphoproteomic analysis, cell lysates were prepared according to specifications from Cell Signaling Technology. Briefly, 2 × 10^8^ cells of each genotype were starved for 2 h in Earl’s Balanced Salt Solution (EBSS; #24010043; Gibco) and then rinsed with ice-cold PBS. After carefully removing all PBS, cells were serially lysed in 10 ml of fresh urea lysis buffer (20 mM HEPES pH 8.0, 9 M urea) supplemented with phosphatase inhibitors (phosStop, Roche), to produce 20–40 mg of protein per condition. Lysates were frozen at −80 °C until processed for PTMScan analysis^77^ (Cell Signaling Technology).

Cellular extracts were sonicated, centrifuged, reduced with DTT, and alkylated with iodoacetamide. In total, 15 mg (antibody enrichment) or 500 μg (IMAC enrichment) total protein for each sample was digested with LysC (antibody) or trypsin (IMAC) and purified over C18 columns for enrichment with the Fe-IMAC (#20432) for total phosphopeptides and Akt substrate motif antibodies (#5561/#5563) for phospho-AGC-motif specific enrichment. Enriched peptides were purified over C18 STAGE tips^78^. Enriched peptides were subjected to secondary digest with trypsin and second STAGE tip prior to LC-MS/MS analysis.

Replicate injections of each sample were run non-sequentially for each enrichment. Peptides were eluted using a 90-min or 150-min linear gradient of acetonitrile in 0.125% formic acid delivered at 280 nL/min. Tandem mass spectra were collected in a data-dependent manner with a Thermo Orbitrap QExactive or Fusion™ Lumos™ Tribrid™ mass spectrometer using a top-twenty MS/MS method, a dynamic repeat count of one, and a repeat duration of 30 s. Real-time recalibration of mass error was performed using lock mass^79^ with a singly charged polysiloxane ion *m*/*z* = 371.101237.

MS/MS spectra were evaluated using SEQUEST and the Core platform from Harvard University^80–82^. Files were searched against the most recent update of the UniProt *mus musculus* FASTA database. A mass accuracy of ±5 ppm was used for precursor ions and 0.02 Da for product ions. Enzyme specificity was limited to trypsin, with at least one tryptic (K- or R-containing) terminus required per peptide and up to four mis-cleavages allowed. Cysteine carboxamidomethylation was specified as a static modification, oxidation of methionine and phosphorylation on serine, threonine, or tyrosine residues were allowed as variable modifications. Reverse decoy databases were included for all searches to estimate false discovery rates, and filtered using a 2.5% FDR in the Linear Discriminant module of Core. Peptides were also manually filtered using a ±5 ppm mass error range and presence of a phosphorylated residue (IMAC) or phosphorylated residue within an RXX(s/t) motif (Akt Substrate Motif antibodies). All quantitative results were generated using Skyline (MacLean) to extract the integrated peak area of the corresponding peptide assignments in the MS1 channel. Accuracy of quantitative data was ensured by manual review in Skyline or in the ion chromatogram files.

### Statistics and reproducibility

All experiments were repeated at least three times. Standard two-tailed Student’s *t* test (when two groups were compared) or one-way ANOVA with Turkey correction for multiple comparisons (where three or more groups were compared) were used in the experiments unless otherwise stated. The relative level of the *p*-value is expressed as asterisks. In general, “ns” = *p* > 0.05, *p < 0.05, **p < 0.01, ***p < 0.001, and ****p < 0.0001.

### Reporting summary

Further information on research design is available in the Nature Research Reporting Summary linked to this article.

## Supplementary information

Supplementary Information

Peer Review File

Supplementary Table 1

Supplementary Table 2

Supplementary Table 3

Supplementary Table 4

Reporting Summary

## Data Availability

Material and correspondence: All data are available from the corresponding author upon reasonable request. The information and requests for resources and materials should be directed to Dr. Mario Pende (mario.pende@inserm.fr). The source data for the phosphoproteome has been deposited to the ProteomeXchange Pride database with accession PXD014832. The source data underlying Figs. 1b–d, f, h, 2a–c, e, g, 3a–d, 4b–d, 5d, 7a–g, 8b–d, and 9b, and Supplementary Figs. 3b, c, 4a, b, 5b, 6a–d, 7b, 9a, 10a–c, 11a, b, d, f, g, and 12c are provided as a Source Data file.

## Source data

Source Data
