# Supplementary material for: mTOR and S6K1 drive polycystic kidney by the control of Afadin-dependent oriented cell division
Source: Nat Commun. 2020 Jun 24;11:3200. doi: 10.1038/s41467-020-16978-z (PMC7314806; doi:10.1038/s41467-020-16978-z)
Supplement: Supplementary file 1 — Supplementary Information [file 41467_2020_16978_MOESM1_ESM.pdf]

Supplementary information

S6K1-dependent polycystic kidney in Tuberous Sclerosis disconnects the controls of cell division rate and orientation by mTOR

Martina Bonucci, Nicolas Kuperwasser, Serena Barbe, Vonda Koka, Delphine de Villeneuve, Chi Zhang, Nishit Srivastava, Xiaoying Jia, Matthew P. Stokes, Frank Bienaimé, Virginie Verkarre, Jean Baptiste Lopez, Fanny Jauli, Marco Pontoglio, Fabiola Terzi, Benedicte Delaval, Matthieu Piel, Mario Pende

Supplementary Figure 1

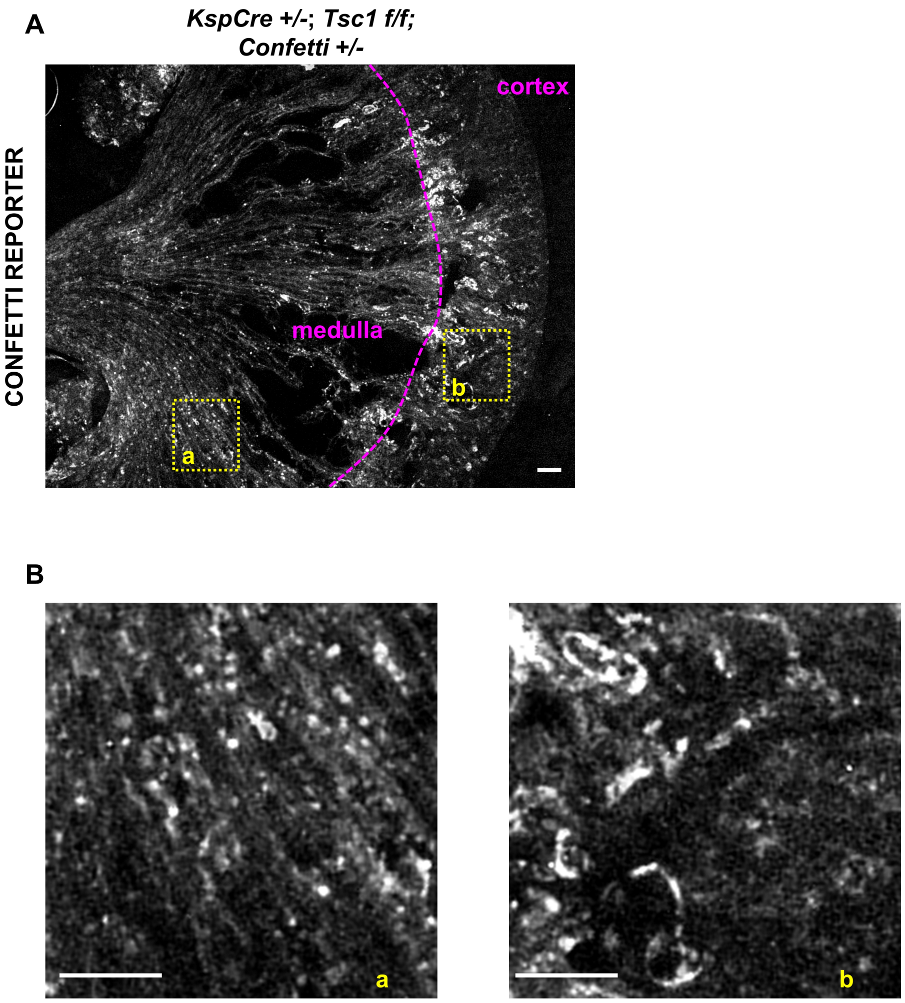

Supplementary Figure 1. Ksp-Cre induces the loss of Tsc1 in both medulla and cortex. (A) Low magnification picture of Ksp-Cre; Tsc1 f/f; Confetti +/- at an early cystic stage (post-natal day 30). All the different colors of the confetti reporters are showed in the false color white. The magenta dotted line shows the border between medulla and cortex. We can observe positive cells in both regions. (B) Insets of picture in (A) showing a higher magnification view of the Confetti-reporter in the medulla (a) and in the cortex (b). Scale bar, 100µm.

Supplementary Figure 2

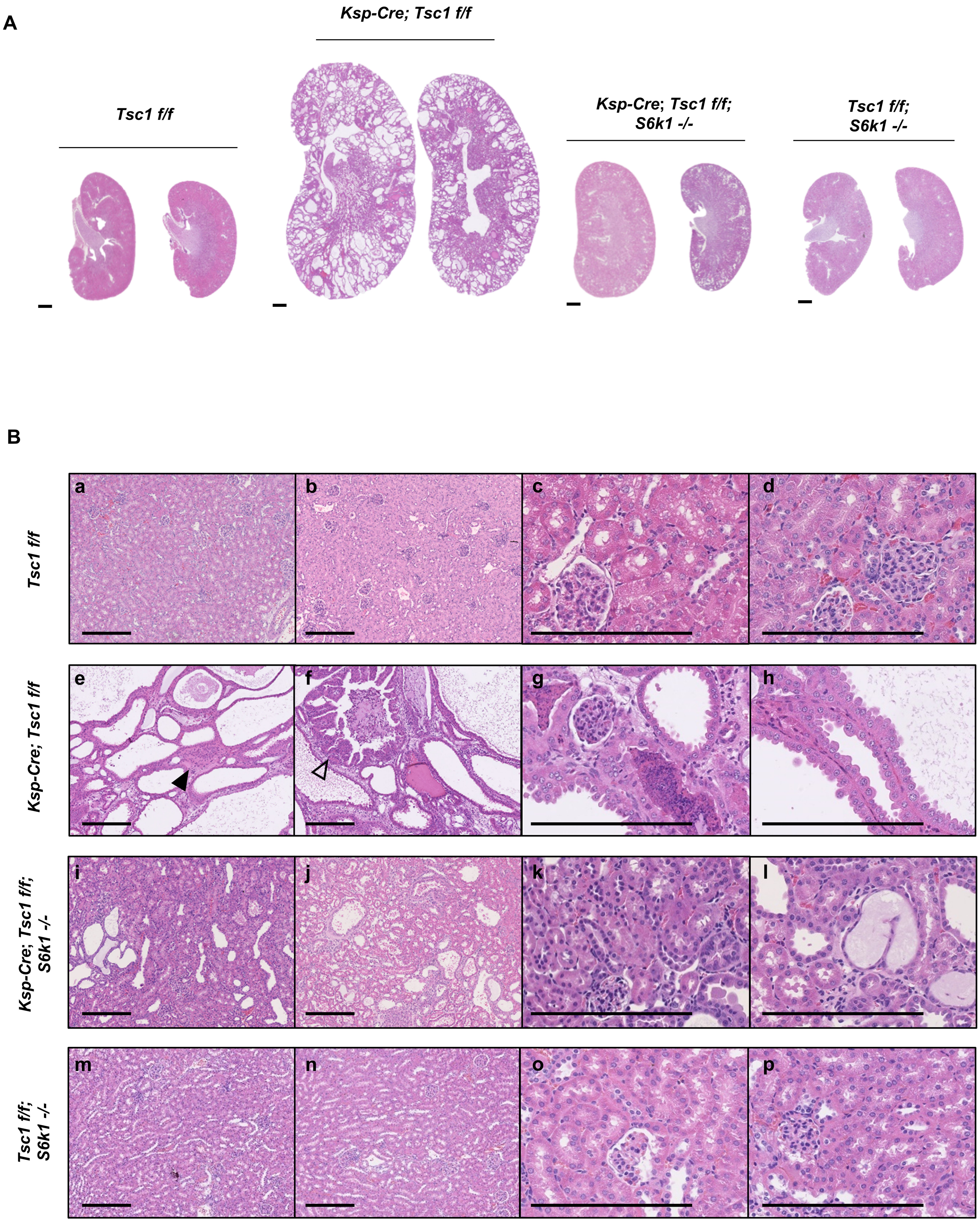

Supplementary Figure 2. *S6k1* deletion improves the TSC-related renal phenotype. (A) Macroscopic photos of Hematoxylin-Eosin (H&E) staining at post-natal day 90 of the different genotypes. Scale bar, 1mm. (B) Images at different magnification of H&E stained samples of post-natal day 90 mice of the different genotypes indicated, to allow a better appreciation of the renal histology and pathology of the different genotypes. Different lesions can be observed in *Ksp-Cre; Tsc1 f/f* animals: in (e) the presence of multiple epithelial cysts and a carcinoma (black arrow); in (f) the presence of epithelial cysts and a cyst-adenoma (empty arrow); in (g) and (h) the typical “columnar epithelium” of the TSC-driven cysts is observable, composed by thicker mis-shaped cells. In (i), (j), (k) and (l) it is possible to observe the decrease in cyst number and size and the decrease of renal lesions, especially cysts, in the *Ksp-Cre; Tsc1 f/f; S6k1 -/-* animals. Scale bar, 250 µm.

Supplementary Figure 3

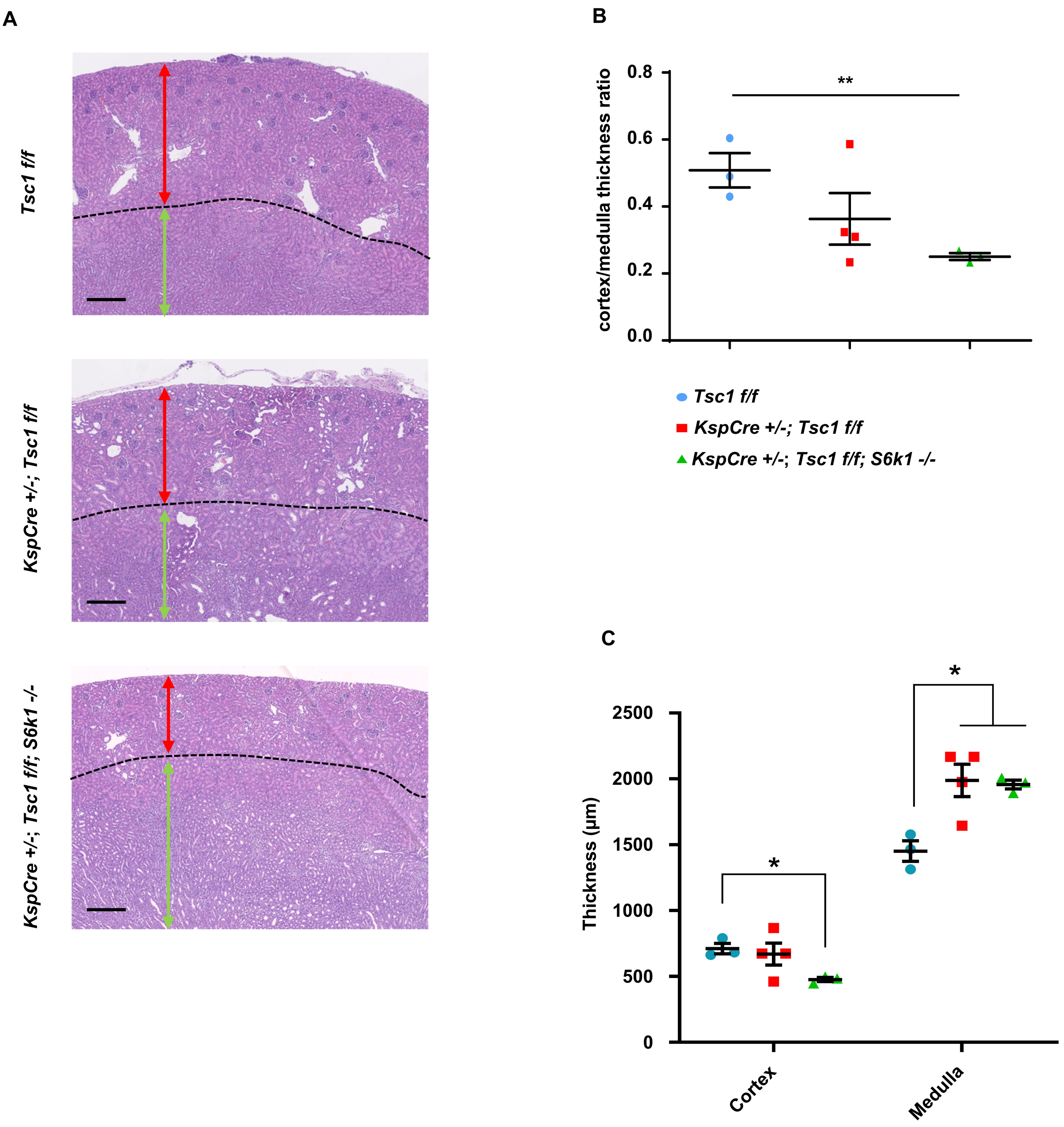

Supplementary Figure 3. The increased proliferation in Tsc1-deleted renal tubules induces their lengthening. (A) Representative images of H&E stained samples of pre-cystic post-natal day 20 mice of the different genotypes. Dotted black lines highlight the separation between cortex and medulla; red and green arrows show the thickness of cortex and medulla respectively. Scale bar, 250 μm. (B) Quantification of the ratio between cortical and medullary thickness in n=3 *Tsc1 f/f*, n=4 *Ksp-Cre; Tsc1 f/f* and n=3 *Ksp-Cre; Tsc1 f/f; S6k1 -/-* mice. Nanozoomer-scanned H&E stained cross sections of kidneys of the different genotypes were analyzed. Both the cortical and the medullary thickness were measured in at least 8 points for each renal section. 8 ratios were calculated for each section, the average was calculated for each animal and plotted in the graph in (B). Mean ± SEM. (C) Absolute values of the thickness of cortex and medulla used to calculate the ratio in (B). Mean ± SEM. The relative level of the p-value is expressed as asterisks. In general, “ns” = p> 0.05, "*" = p< 0.05, "**" = p< 0.01, "***" = p< 0.001, and "****" = p<0.0001.

.

Supplementary Figure 4

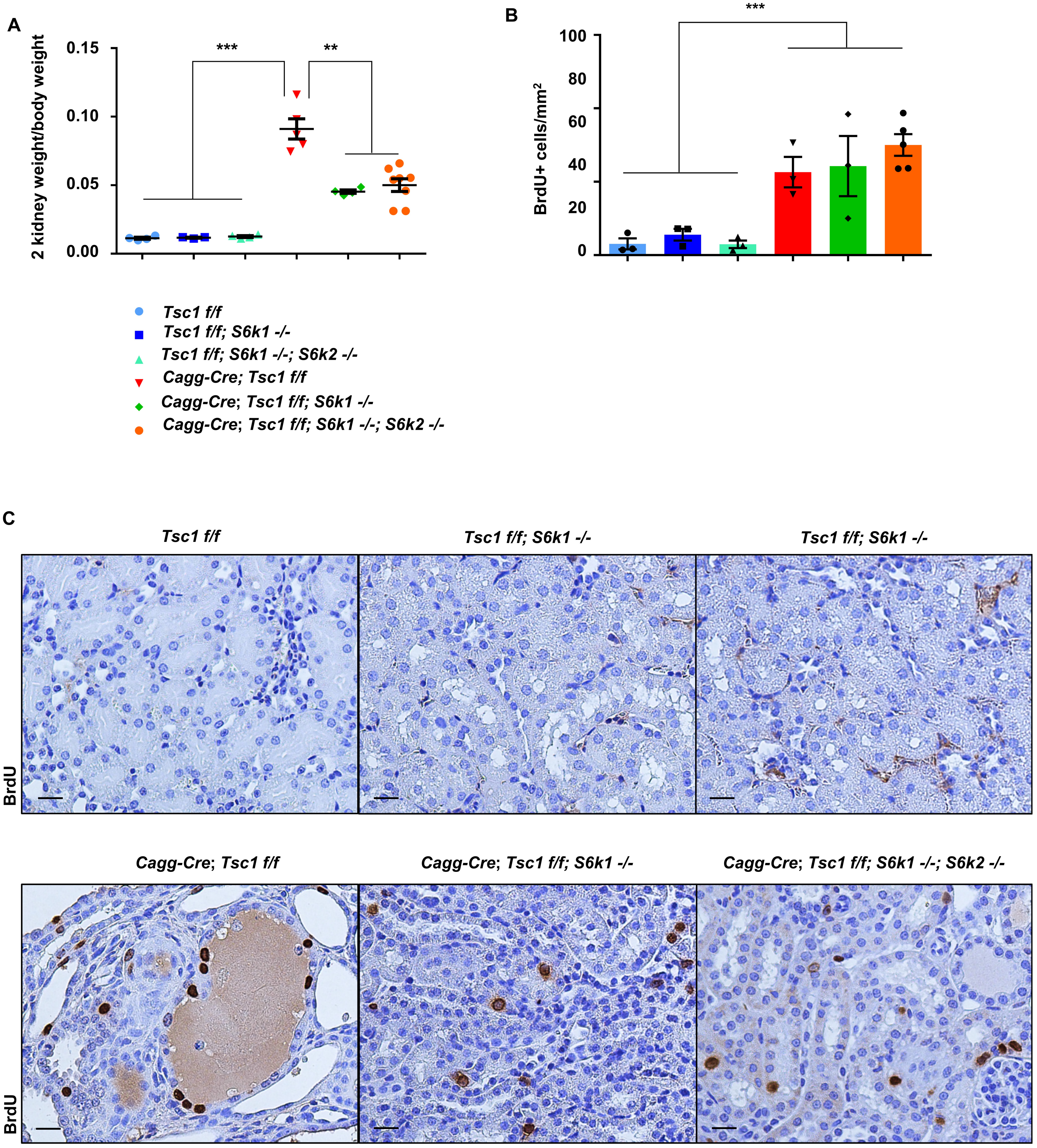

Supplementary Figure 4. S6K1 and not S6K2 is responsible for the cystic phenotype. (A) Kidney/body weight ratio of the indicated phenotypes at post-natal day 90. Mean ± SEM. n=4 *Tsc1 f/f*, n=3 *Tsc1 f/f; S6k1 -/-*, n=4 *Tsc1 f/f; S6k1 -/-; S6k2 -/-*, n=5 *Cagg-Cre; Tsc1 f/f* and n=4 *Cagg-Cre; Tsc1 f/f; S6k1 -/-,* n=8 *Cagg-Cre; Tsc1 f/f; S6k1 -/-; S6k2 -/-* mice were analyzed. (B) Quantification and (C) representative images of BrdU staining at post-natal day 90 of the different genotypes indicated. The percentage of BrdU-positive cells per mm^2 was counted in 10 different fields of each section. Mean ± SEM. n= 3 mice/group. Scale bar, 20µm. The relative level of the p-value is expressed as asterisks. In general, “ns” = p> 0.05, "*" = p< 0.05, "**" = p< 0.01, "***" = p< 0.001, and "****" = p<0.0001..

Supplementary Figure 5

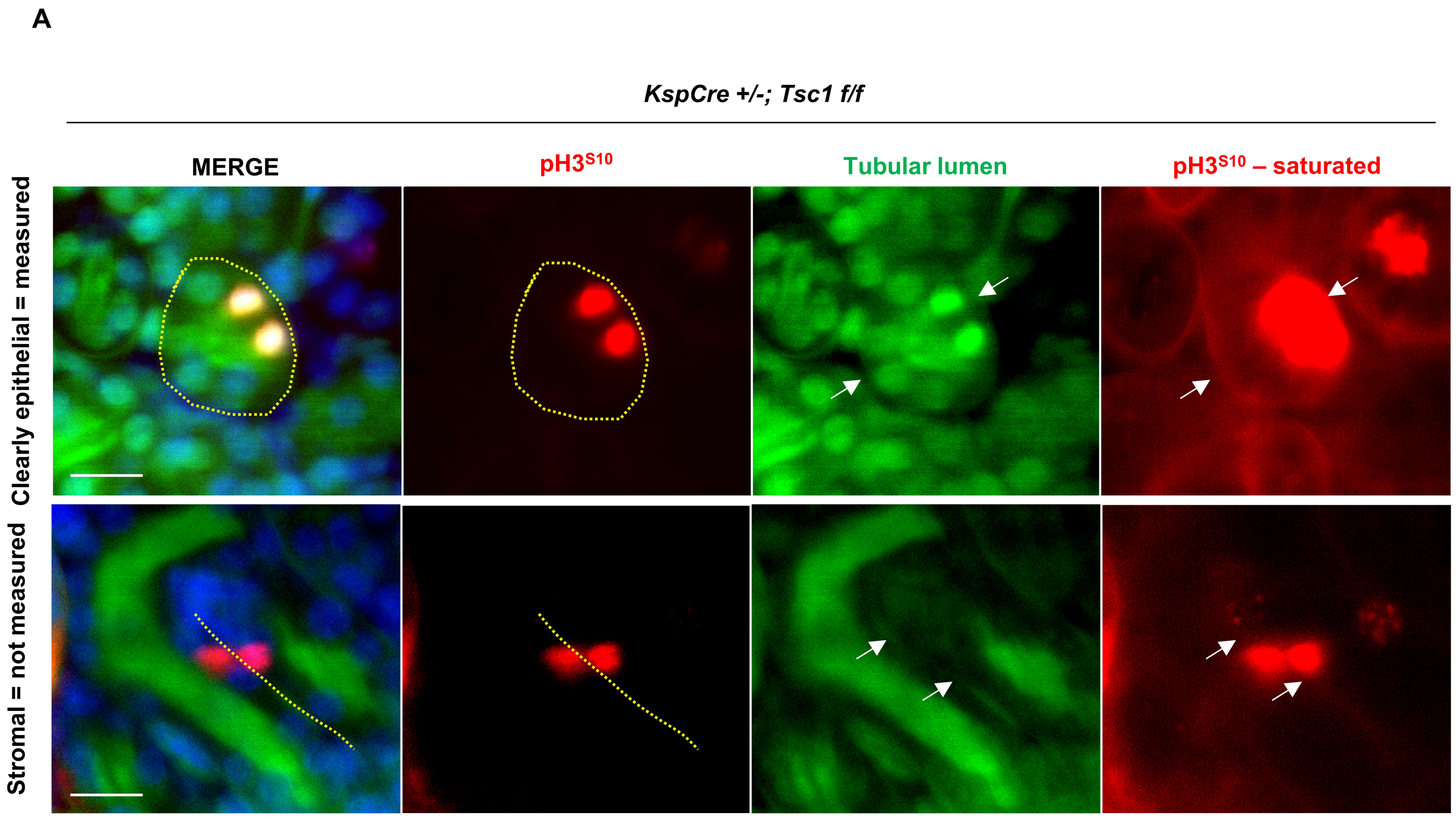

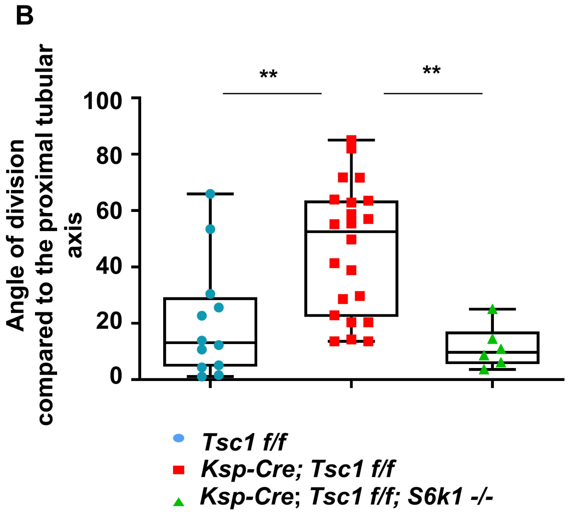

Supplementary Figure 5. Discrimination of tubular vs. stromal mitosis in cleared images. (A) Examples of pictures of cleared and stained kidneys of *Ksp-Cre; Tsc1 f/f* animals, as in Fig. 2F. The mitotic divisions are in red and the tubular axis in green, before digital 3D reconstruction. Yellow dotted lines represent the basal borders of the renal tubules, visualized by increasing the display intensity of the signal with either green or red. The white arrow are positioned to compare the green and red saturated signal. Both signals highlight the presence of the basal borders in overlapping positions. The upper panels show an example of mitosis clearly in an epithelial cell and, therefore, included in the quantifications. The lower panels show a mitosis that doesn't belong to a tubular epithelial cell, because it crosses the limits of the basal tubular border. Mitosis not clearly in the tubules, as in the lower panel, were not included in our study. Scale bar, 20 µm. (B) Quantification of the mitotic division orientation at post-natal day 20 in distal tubules of *Tsc1 f/f* (n=12), *Ksp-Cre; Tsc1 f/f* (n=22) and *Ksp-Cre; Tsc1 f/f; S6k1 -/-* (n=6) mice. To assess the significance of the data, a Mann-Whitney U test was used. Median ± interquartile range and Min and Max values. The relative level of the p-value is expressed as asterisks. In general, “ns” = $p > 0.05$, "*" = $p < 0.05$, "**" = $p < 0.01$, "***" = $p < 0.001$, and "****" = $p < 0.0001$.

Supplementary Figure 6

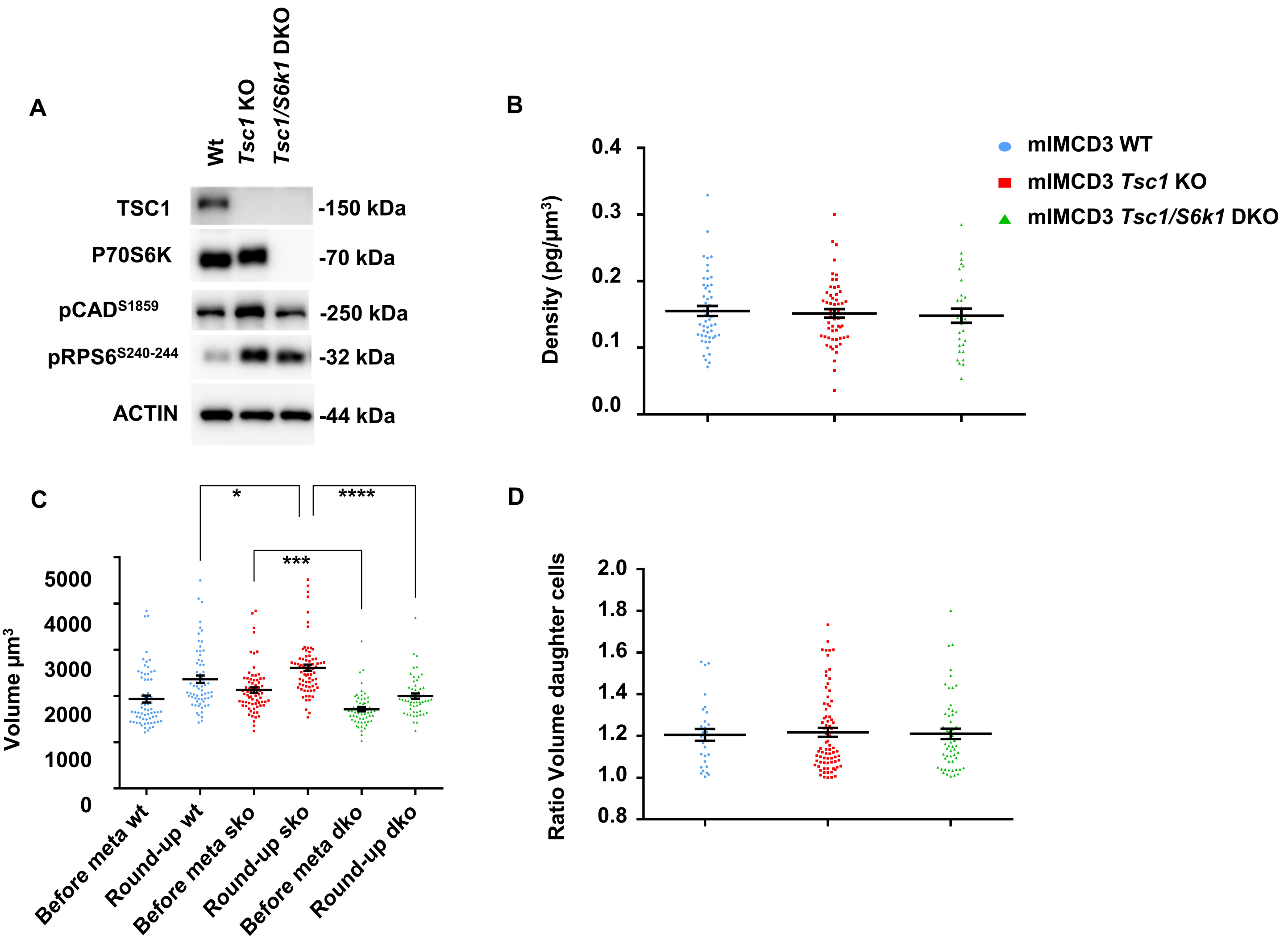

Supplementary Figure 6. Characterization of CRISPR/Cas9-mediated deletion of *TSC1* and *S6K1* in mIMCD3 cells. (A) mIMCD3 cells were mutated for either *Tsc1* or *Tsc1/S6k1* by CRISPR/Cas9. After selection, cells were plated and protein extracts were tested for the expression of TSC1 and S6K1 proteins and for targets of the activation of the pathway. (B) Cell density was analyzed as the ratio of mass to volume measured for the same cells by optical measurement at G1 (2.5 h after mitotic division) for the different genotypes. An average of 40 cells pooled from 3 independent experiments were quantified for each genotype. Horizontal lines represent the mean ± SEM. (C) With the same optical measurement, the volume of cells in mitosis was analyzed. The volume was measured just before metaphase round-up and during the metaphase round-up for the different genotypes. An average of 60 cells pooled from 3 independent experiments were quantified for each genotype. Horizontal lines represent the mean ± SEM. (D) The volume of two daughter cells after mitosis was analyzed to calculate the relative ratio. A minimum of 100 cells pooled from 3 independent experiments were quantified for each genotype. Horizontal lines represent the mean ± SEM. The relative level of the p-value is expressed as asterisks. In general, "ns" = $p > 0.05$, "*" = $p < 0.05$, "**" = $p < 0.01$, "***" = $p < 0.001$, and "****" = $p < 0.0001$.

Supplementary Figure 7

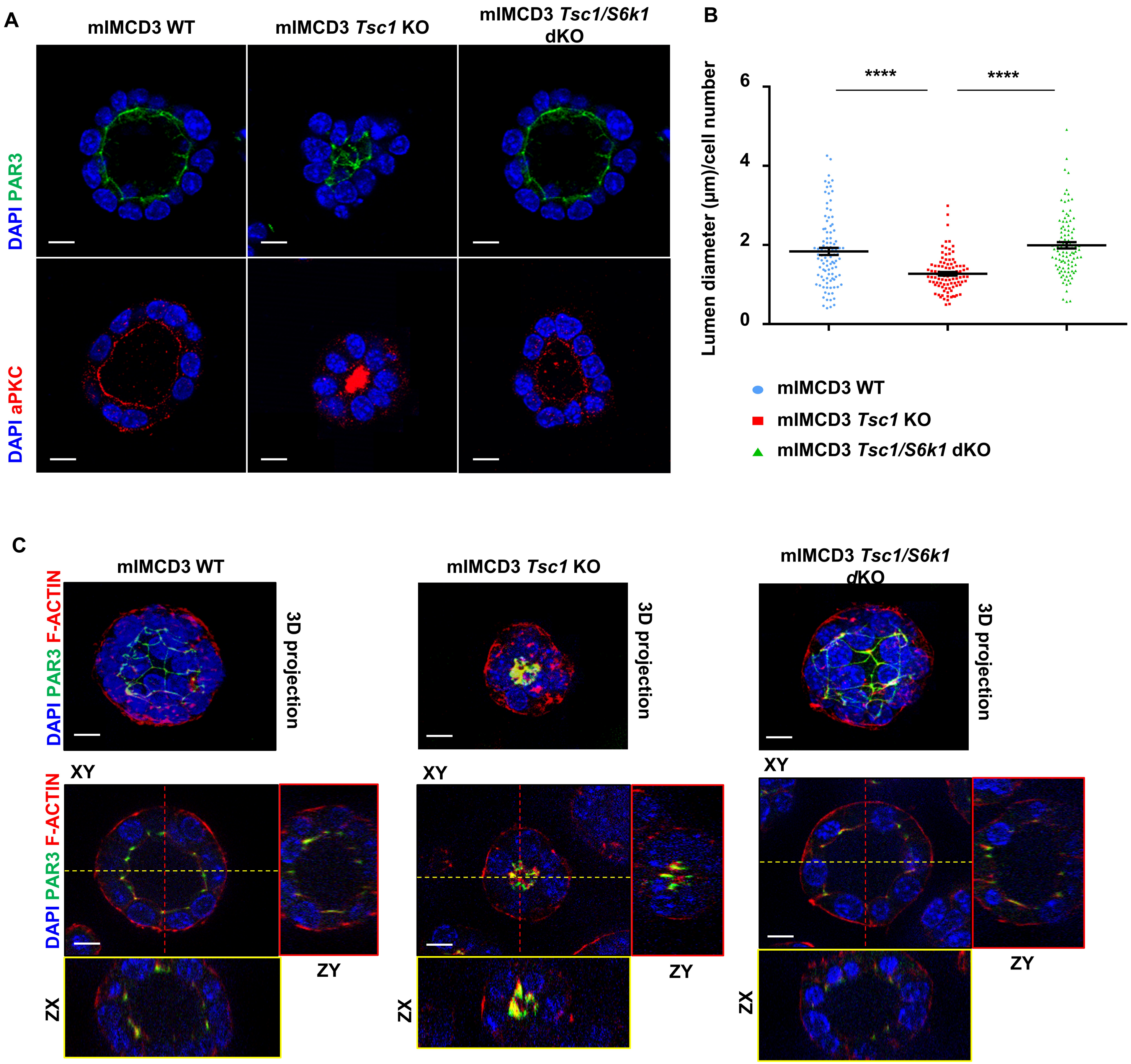

Supplementary Figure 7. The lumen of *Tsc1* KO spheroids are correctly located but fail to enlarge. (A) Representative images and (B) quantification of the ratio between the diameter of the lumen of 5-day spheroids at their central z-plane, divided by the number of nuclei surrounding the lumen in the same z-plane. The ratio is expressed in µm/number of cells. The lumen was stained by apical markers PAR3 (green) or aPKC (red). An average of 100 spheroids pooled from 3 independent experiments were quantified for each genotype. Horizontal lines represent the mean ± SEM. Scale bar, 10 µm. (C) 3D projections (top) and relative orthogonal projections (bottom; XY, ZY and ZX orthogonal planes displayed) of 5-day spheroids immunolabelled with PAR3 and Phalloidin to stain the apical and the basal side of the spheroids for each indicated genotype. In the three genotypes it is possible to observe luminal presence of PAR3, although the size of the lumen in the Tsc1 KO spheroids is decreased. Scale bar, 10 µm. The relative level of the p-value is expressed as asterisks. In general, “ns” = $p > 0.05$, "*" = $p < 0.05$, "**" = $p < 0.01$, "***" = $p < 0.001$, and "****" = $p < 0.0001$.

Supplementary Figure 8

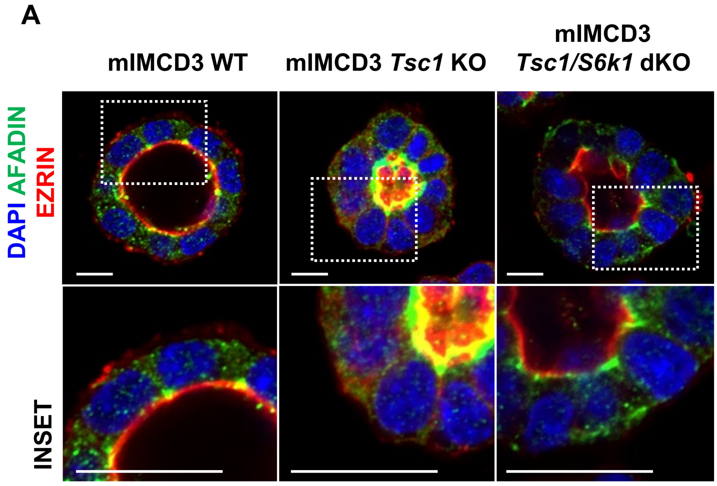

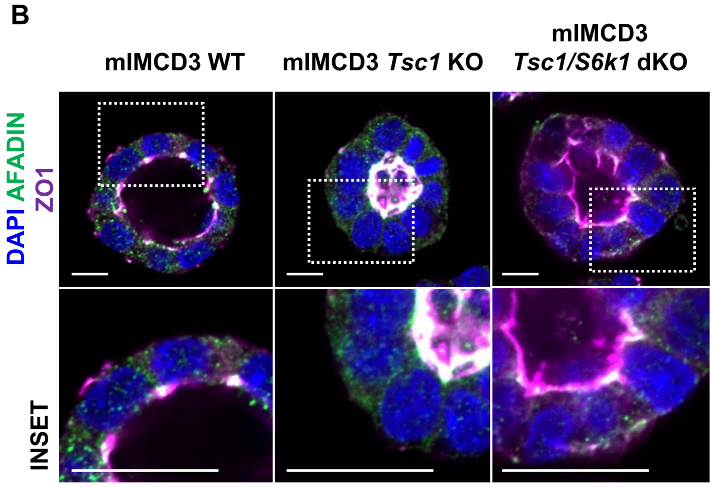

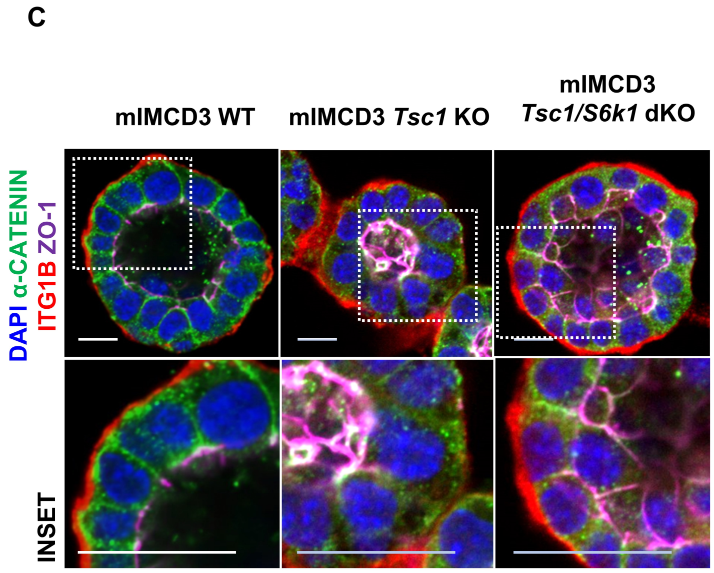

Supplementary Figure 8. Apico-basal polarity is maintained in *Tsc1* KO spheroids. (A) and (B) Representative images and insets of 5-day mIMCD3s spheres of the indicated genotypes immunolabelled with Afadin (green), Ezrin (red) and ZO-1 (magenta), to visualize and compare the localization of the apical domain - Ezrin, the tight junctions - ZO-1, and the adherens junctions – Afadin. (A) Represents the merge of Afadin and Ezrin staining; (B) represents the merge of Afadin and ZO-1. Note the strong degree of colocalization of the three distinct apical domains in the Tsc1 KO spheroids, compared to the other two genotypes. Scale bar, 10 μm. (C) Representative images and insets of 5-day mIMCD3s spheres of the indicated genotypes immunolabelled with α-catenin (green), ZO-1 (magenta) and Integrin-β1 to visualize the baso-lateral, apical and basal domains respectively. White staining indicates co-localization of magenta and green staining. Note the loss of baso-lateral staining and the concomitant apical accumulation of α-catenin in the Tsc1 KO spheroids, compared to the other two genotypes. The signal intensity of Integrin-β1 has been standardize to show the localization; however, slight differences in the intensity were present between the genotypes, notably with decrease in the Tsc1 KO and Tsc1/S6k1 dKO. Scale bar, 10 μm.

Supplementary Figure 9

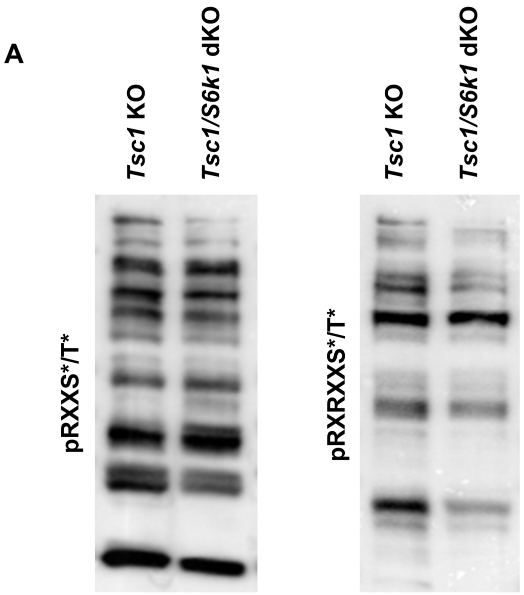

Supplementary Figure 9. Pattern of phosphorylated proteins with AGC-recognition motif in *Tsc1* KO vs. *Tsc1/S6k1* dKO cells. (A) Western blot of total cell extracts from the indicated genotypes, collected after 1.5h starvation in EBSS. Membranes were probed with two different AGC kinase-motif antibodies (anti-RXXS*/T* or anti-RXRXXS*/T*) used in combination in the phosphoproteomic screening.

Supplementary Figure 10

A

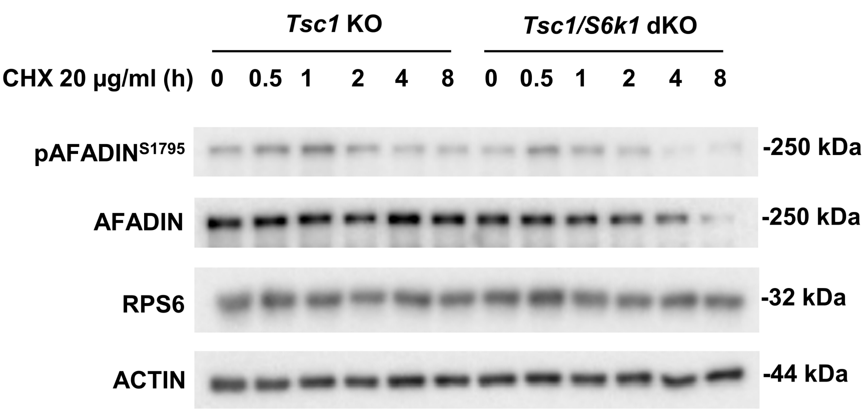

B

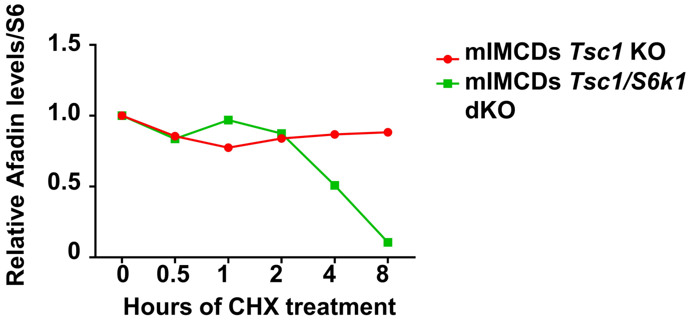

C

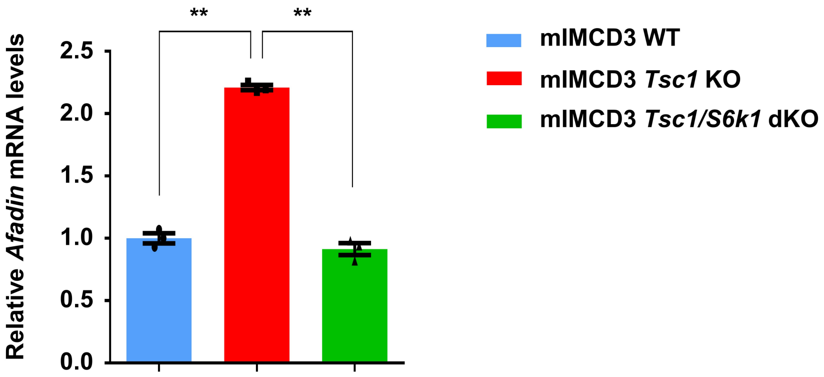

Supplementary Figure 10. S6K1 activity affects both the transcription and the stability of Afadin. (A) *Tsc1* KO and *Tsc1/S6k1* dKO mIMCD3s were plated and treated at the same time with 20 µg/ml of cycloheximide (CHX) in normal medium, to block protein translation. Cells were then collected at the different times indicated and protein lysates were probed with the indicated antibody to compare the stability of Afadin in the two genotypes. (B) Densitometry analysis and quantification of the Afadin amount during CHX treatment relative to RPS6 total protein. (C) RT–QPCR for *Afadin* expression from mIMCD cells of the indicated genotypes. Data are normalized to the control WT cells and are an average of 3 independent experiments. Mean ± SEM. The relative level of the p-value is expressed as asterisks. In general, “ns” = p> 0.05, "*" = p< 0.05, "**" = p< 0.01, "***" = p< 0.001, and "****" = p<0.0001.

Supplementary Figure 11

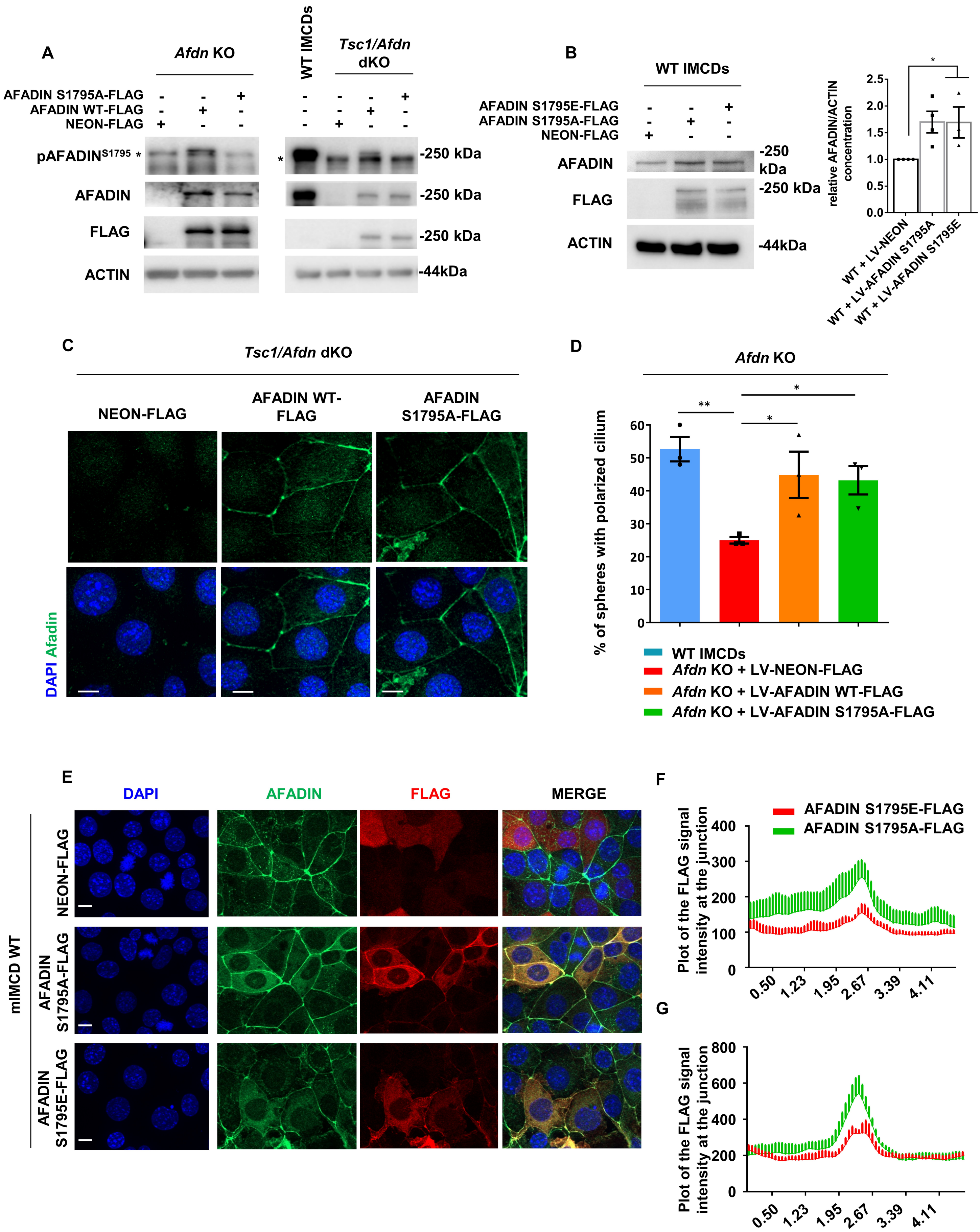

Supplementary Figure 11. Effects of Afadin phospho-mutants on sphere and junctions formation. (A) Cells *Afdn* KO and *Tsc1/Afdn* dKO were transduced with lentivirus FLAG-Neon, FLAG-Afadin-WT or FLAG-Afadin-S1795A. Cells were plated, then serum starved overnight and subsequently amino acid starved with EBSS for 2h. They were then collected and cell lysates were probed with the indicated antibodies. * represents a non-specific cross-reactive band. (B) WT cells were transduced with lentivirus FLAG-Neon, FLAG-Afadin-S1795A or FLAG-Afadin-S1795E. Cells were plated, then serum starved overnight and subsequently amino acid starved with EBSS (Earle's Balanced Salt Solution) for 2h. They were then collected and cell lysates were probed with the indicated antibodies. The graph on the right shows the densitometry analysis and quantification of the Afadin amount in the different genotypes on the levels of actin. The histograms are an average of 4 independent experiments normalized on the FLAG-Neon transduced-derived spheres. Mean ± SEM. (C) *Tsc1/Afdn* dKO cells were plated and probed with anti-Afadin antibody to confirm the correct localization of the constructs. Scale bar, 10 µm. (D) *Afdn* KO cells were transduced with lentivirus FLAG-Neon, FLAG-Afadin-WT or FLAG-Afadin-S1795A. Quantification of the percentage of spheres with polarized primary cilia. The ratio was calculated on roughly n=70 spheres per experiment. Histograms are an average of 3 independent experiments. Mean ± SEM. (E) The WT cells were transduced with lentivirus FLAG-Neon, FLAG- Afadin-S1795A or FLAG-Afadin-S1795E and probed with anti-Afadin antibody and anti-FLAG antibody to check the localization of the constructs. Yellow arrows point at weak junctions in FLAG-Afadin-S1795E transduced cells. Scale bar, 10 µm. (F) and (G) show the plots of the signal intensity at the cell-cell junctions for FLAG and Afadin signals respectively. Different junctions were analyzed per genotype with ImageJ software. The intensity of the signals was quantified along a 5µm long, 50-pixels thick line perpendicular to the junction. The intensity over distance was plotted and the mean for each genotype was calculated. n=4 cells per genotype were analyzed. Mean ± SEM for each micron. The relative level of the p-value is expressed as asterisks. In general, “ns” = p> 0.05, "*" = p< 0.05, "**" = p< 0.01, "***" = p< 0.001, and "****" = p<0.0001.

Supplementary Figure 12

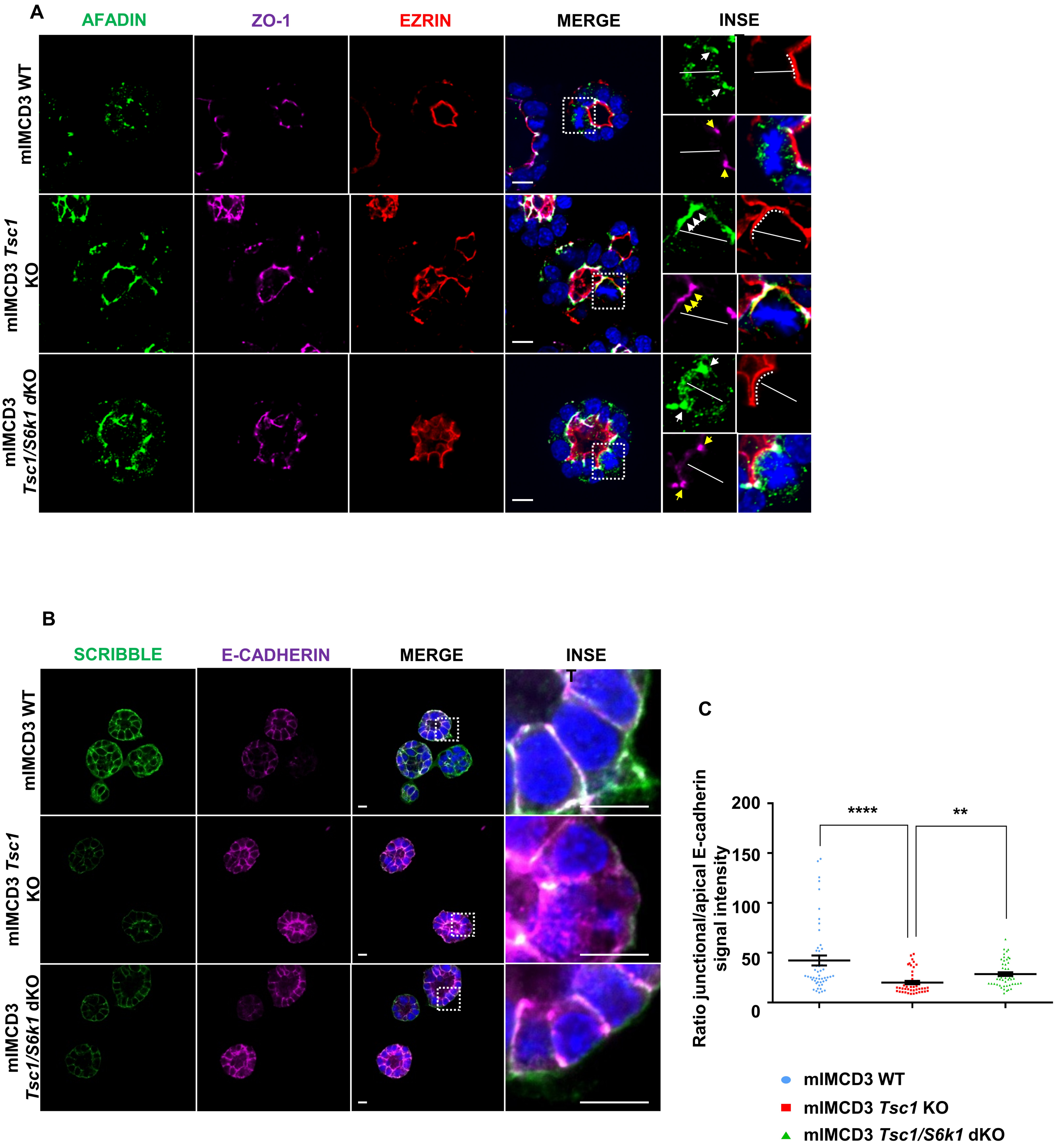

Supplementary Figure 12. *Tsc1*-deficient spheres display altered Afadin/Cadherin-based adhesion system localization. (A) Representative images of 5-day mIMCD3s spheres of the indicated genotypes immunolabelled with Afadin (green), ZO-1 (magenta), Ezrin (red) antibodies and DAPI to show the relative localization of the distinct apical domains in interphase and relative to the mitotic spindle poles in 3D spheroids. Insets show higher magnification of one mitotic cell per genotype. In the insets, yellow arrows indicate the localization of the Afadin spots and white arrows the localization of ZO-1 poles in metaphase cells. A white dotted line represents the mitotic equator. A yellow dotted white line indicates Ezrin staining in the whole apical domain of mitotic cells. Scale bar, 10 μm. (B) Representative images and insets of 5-day mIMCD3s spheres of the indicated genotypes immunolabelled with E-cadherin, Scribble and DAPI to show the loss of baso-lateral presence of both E-cadherin and Scribble in the Tsc1 KO, with a concomitant appearance of an apical aberrant localization for both proteins, as highlighted in the inset. Scale bar, 10 μm. (C) Quantification of the presence of junctional over apical E-cadherin staining in 3D spheroids of the indicated genotypes. E-cadherin intensity at the junctional and apical membrane was measured as the difference (Δ) between the maximal and the minimal value of the plot of the intensities in a 30-pixels thick line perpendicular to the cell-cell junction (junctional pool) or to the cell-lumen surface (apical pool). Each dot represents the average of the ratio between the junctional over the apical E-cadherin intensities measured in each cell of a spheroid at its central z-plane. An average of 50 spheroids pooled from 3 independent experiments were quantified for each genotype. Horizontal lines represent the mean ± SEM. The relative level of the p-value is expressed as asterisks. In general, “ns” = p> 0.05, "*" = p< 0.05, "**" = p< 0.01, "***" = p< 0.001, and "****" = p<0.0001.

Supplementary Figure 13

A

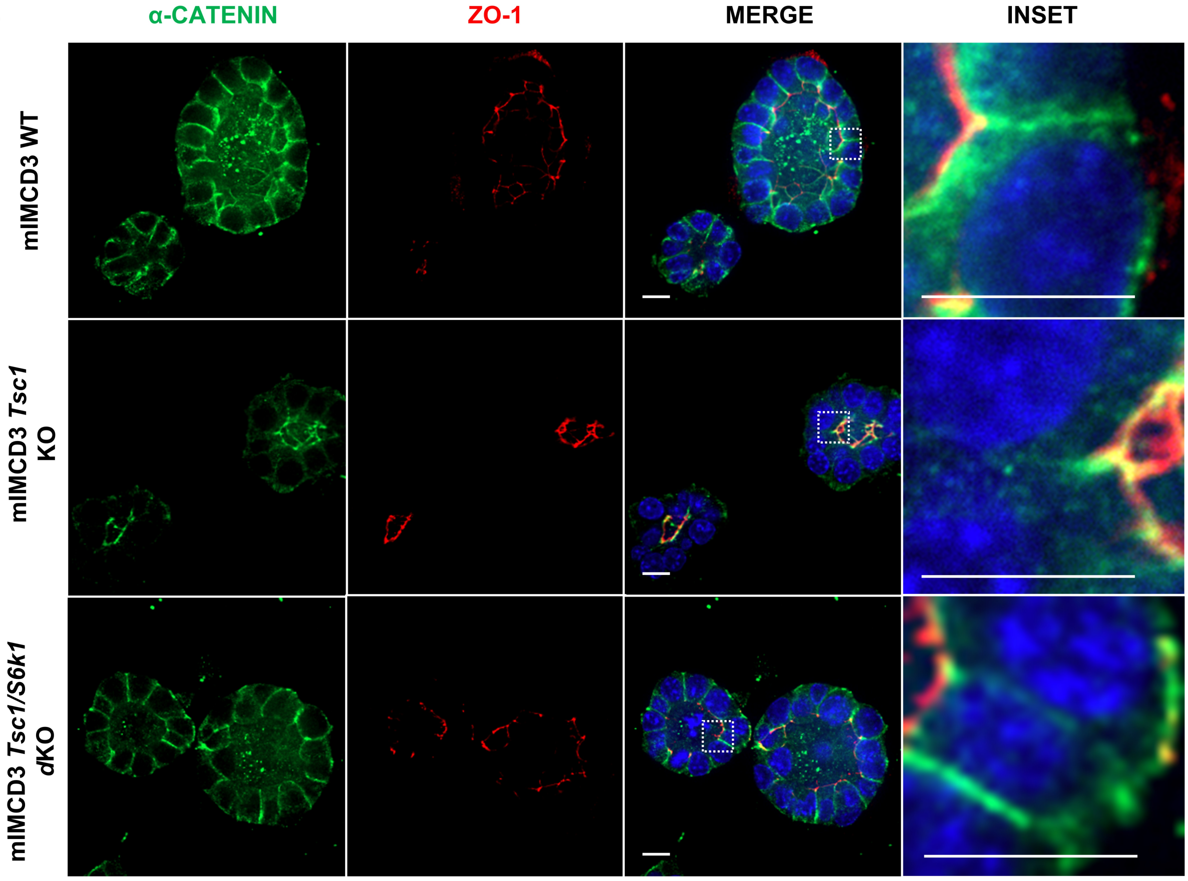

B

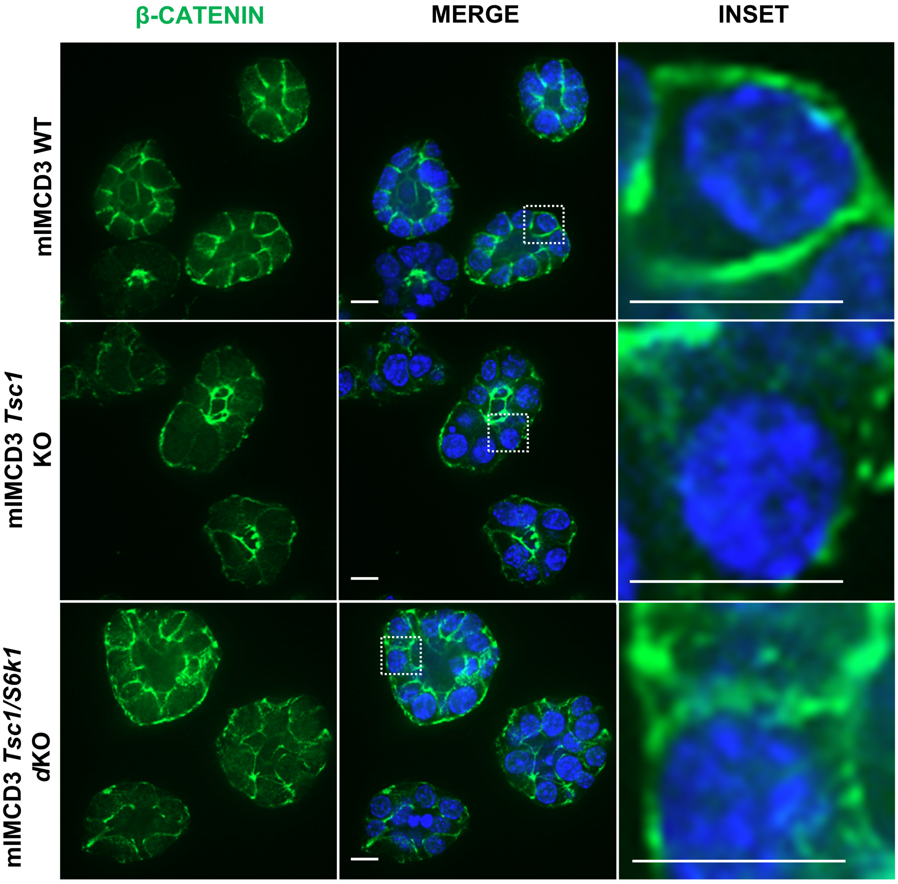

Supplementary Figure 13. *Tsc1*-deficient spheres display altered catenin-based adhesion system localization. (A) Representative images and insets of 5-day mIMCD3s spheres of the indicated genotypes immunolabelled with α-catenin (green) and ZO-1 (red) antibodies and DAPI to show the apical mislocalization of α-catenin in Tsc1 KO spheroids compared to the other two genotypes. Scale bar, 10 µm. (B) Representative images and insets of 5-day mIMCD3s spheres of the indicated genotypes immunolabelled with β-catenin (green) antibody and DAPI to show the apical mislocalization of β-catenin in Tsc1 KO spheroids compared to the other two genotypes. Scale bar, 10 µm.
