# Supplementary material for: mTOR and S6K1 drive polycystic kidney by the control of Afadin-dependent oriented cell division
Source: Nat Commun. 2020 Jun 24;11:3200. doi: 10.1038/s41467-020-16978-z (PMC7314806; doi:10.1038/s41467-020-16978-z)
Supplement: Supplementary file 2 — Peer Review File [file 41467_2020_16978_MOESM2_ESM.docx]

Peer Review Information

**Journal:** Nature Communications

**Manuscript title:** mTOR and S6K1 drive polycystic kidney by the control of afadin-dependent oriented cell division

**Corresponding author name(s):** Dr Mario Pende

**Editorial notes:**

**none**

**Reviewer comments & decisions:**

**Reviewer comments, first version:**

Reviewers' comments:

Reviewer #1 (Remarks to the Author):

In the manuscript “S6K1-dependet polycystic kidney in Tuberous Sclerosis disconnect the controls of cell division rate and orientation by mTor” the authors propose a novel hypothesis that renal cystogenesis mediated by lack of mTor occurs via increased S6K1 activity, which promotes abnormal oriented cell division that, when combined with hyperproliferation, causes cystogenesis. They identify S6K1 as a direct mTORC1 substrate. The loss of Tsc causes defects in OCD and loss of both Tsc and S6K1 rescues the cystogenesis and OCD. S6K1 deletion affects the orientation but not the rate of cell division in TSC mutant mice. They go on to show that S6K1 phosphorylates Afadin, an adherens junction/tight junction protein known to be involved in junction formation and OCD. In Tsc1 mutants, there is increased level of Afadin and phospho-Afadin. They show that mutation of Afadin at the phosphorylation site rescues the Tsc1 mutants, and conclude that increased phospho-Afadin is what leads to the misoriented OCD.
Overall this manuscript has some really interesting data that advances the field and the experiments are well done. However, there are some points that need to be supplemented with additional experimentation to truly support their conclusions. Please see the following points:

Major points
1) Fig 2. Was the spindle angles measured in all tubules? Did they mainly examine the cortex? This is not stated in the methods section. This is important because Ksp-cre recombines in only ~5-10% of proximal tubules, and some of these abnormal measurements could be due to non-cell autonomous defects. Please clarify and address this point.
2) Fig 4. A cyst that does not have a clear central lumen (i.e. clear demarcation of apical and basal) cannot be used to assess spindle angle, as they do for the TSC knockouts in Figure 4. This is because there is no true frame of reference.
3) The authors show that the TSC1 KO cells have more Afadin and more phospho-Afadin than wild type and TSC/S6K1 KO cells. Their data demonstrates that S6K1 phosphorylates Afadin, and they suggest that the phosphorylation promotes Afadin protein stability. However, this can (and should) be tested directly in vitro to support or refute this claim.
4) Also, if it is increased phospho-Afadin that promotes misoriented OCD, then overexpression of a phospho-mimetic at this site in wild type would be expected to have the same effect. This is a critical experiment that should be done.
5) In Fig 8, they report that afadin staining is increased at the apical/lumenal side in TSC mutants compared with control. This experiment needs to be done in conjunction with an apical marker and with a junctional marker (e.g. ZO1) because some of the afadin that appears apical can in fact be apical junction due to the plane being visualized. Also, is the phospho-Afadin at the apical site?
6) In Fig 8 the authors report that the junctions are mispositioned. However, if there is no normally-located lumen, it is not clear that the junctions are the issue. Also, Ecadherin appears globally reduced, even at the later borders of cells, based on the IF. The location of Ecadherin at these sites it not expected to be altered by changes in afadin, which is normally restricted to the apical-junctional complex. Please address.
7) In the discussion they report that “Centrosome misposition in mitotic cells underlies the defect in oriented cell division”. The data suggests the two are correlated in that both are abnormal, but the data does not show any causality. Thus, the conclusions are not supported by the results.
8) Loss of Afadin has been shown to cause misoriented cell division in vitro and in vivo in renal tubules. The authors should cite this early in the manuscript, as it is central to the theme and not mentioned until nearly the end of the discussion. How do the authors explain that loss of Afadin causes misoriented cell division in the other study and that increased expression of phospho-Afadin causes causes loss of oriented cell division in their study?

Minor point
1) Fig S1: please plot data in columns as individual values rather than mean/median, as you do in Fig 1.


Reviewer #2 (Remarks to the Author):

This is an interesting paper from Bonucci … Pende on observations on renal cyst formation in mouse models of TSC. The findings are novel, and will be of general interest to the community of TSC and mTOR investigators.
However, there are several issues.

Major:
1. Figure 1A. It appears that there are small cysts in the Ksp-Cre; Tsc1 f/f; S6k1 -/- kidneys. A Supplemental Figure should be made with both medium and high power images from all genotypes, multiple panels from different kidneys, to give the reader a full appreciation of histology and pathology in these mouse kidneys.
2. What happens to the increased number of cells generated by division in the Tsc1KO S6K1 KO kidneys. Is there a net gain in the number of cells without expansion of the kidney size? Seems a bit odd. Can you quantify total cell counts in those kidneys? This may connect with requested high power views of the kidneys in 1, above. Note that Figure E and G left panels, controls, are not adequate, as they do not show normal renal tubules, 1G left is substandard, a washed out image as well.
3. Figure 2A should include a Tsc1 blot.
4. For both the ksp-cre and CAGG-cre models, it seems that not all cells are dividing at any time. Why not?
5. Figure 2E and F. This needs to be demonstrated better. Add S figure to show many of these cells captured in mitosis by this technique. In addition, n=30 mitotic events is not enough, please do at least 100 each. Why are so few mitoses at an angle near 0° in the control mice?
6. ‘The phosphorylation of ribosomal protein S6 (RPS6) was not completely inhibited in S6K1 deficient kidneys, owing to the presence of S6K2’ Is there evidence of this from previous studies, or in work the authors can show? Figure 2A shows complete absence of S6K in the S6K1-/- kidneys, suggesting there is no expression of S6K2.
7. My eye sees that pAKT levels are pretty similar across Figure 2A blot, in contrast to the statement in the text.
8. Figure 4C top does not look like the other images of Tsc1 KO/S6k1 KO cells. The cells are bunched up and not forming a nice spheroid. Please generate some quantitative or semi-quantitative measure of spheroid formation, e.g. lumen to cell # ratio, and measure this for large numbers of spheroids, to provide quantitative data for each genotype.
9. Figure 6 A label in the figure says AKT volcano plot while the legend says AGC kinase-motif. This figure goes out of focus when seen at high enough mag to read the labels. Please add in the image if red means up in which genetic mix vs. which other one. The figure legend is also not clear on this point; green means up in Tsc1 KO vs. Tsc1 KO/S6K1 KO?
10. Was there only one phosphorylation site for afadin identified in the mass spec analysis? Multi-site phosphorylation is common.
11. The images in Figure 8C are crucial, and are not well-explained. What are all the small squares with arrows and some colors? It seems that the columns of figures are different examples for each genotype, but this is not clear. The legend does not explain well either. Why is there so much variability, some cells seem to express no E-cadherin at all, while in others it is abundant?
12. Please do IHC on the mouse kidneys, all 3 genotypes, to show afadin and p-afadin localization in both resting and mitotic cells.

Minor comments:
1. ‘In addition, TSC patients display increased risk to develop polycystic kidney disease, which may represent an important morbidity and mortality factor.’ This is an overstatement. 2-3% of TSC patients develop severe PKD due to concurrent genomic mutation in TSC2 and PKD1. Other TSC patients have kidney cysts, but they are few in number, and not a source of morbidity or renal dysfunction.
2. ‘mediate cyst formation are elusive.’ Prefer unknown rather than elusive. No one has looked before, does not mean they are elusive.
3. ‘phosphorylation of other mTORC1 substrates may be relatively spared’ There is quite good evidence from past studies from the Sabatini lab that sensitivity of mTORC1 to rapalog inhibition varies a great deal among substrates with S6K1 being the most sensitive.
4. Further suggestive evidence that S6Ks are driving cyst formation is the sensitivity of cyst formation in various genetic models to rapalog therapy, given the sensitivity of S6K phosphorylation to rapalogs, and relative resistance of other substrates.
5. ‘plasmatic urea levels’ should be serum urea levels; at least that is the usual blood derivative used for urea measurement.
6. ‘a net amelioration of’ replace with ‘improvement in’
7. ‘up-regulates the rate of cell proliferation, though the tubular cells are protected against tubular enlargement.’ Prefer ‘increases cell proliferation but cyst formation does not occur.’
8. ‘a major determinant of cyst formation’ prefer ‘required for robust cyst formation’.
9. Mouse gene names (re: mIMCD3 cells) should be italicized with first letter only in caps. E.g. Tsc1 for mouse, not TSC1.
10. ‘with an average of around 16 cells corresponding to 5 doubling times’ 2**4=16, hence 16 cells corresponds to 4 doubling events or sets of cell division.
11. ‘In human clinical trials, rapamycin derivatives clearly improved kidney function in TSC patients 69, while the results against ADPKD were moderate or negative 70, 71, 72.’ This statement is misleading. The benefit of rapamycin in TSC kidneys is mainly due to reduction in size of angiomyolipoma, leading to improved kidney function. Angiomyolipomas do not occur in ordinary ADPKD.
12. It’s hard to appreciate the magnitude of the difference in the red vs. other color dots in Fig 3CD. Please figure out a presentation method that makes this easier to see.


Reviewer #3 (Remarks to the Author):

Bonucci et al. Nat Comm 2019

The authors have made the surprising discovery that mTORC1 regulates cell proliferation via an S6K1-independent pathway, while an S6K1 dependent pathway regulates the orientation of cell division (OCD), in part via its substrate Afadin, which is involved in cell adhesion.

In Figure 1, Ksp-Cre; Tsc1 f/f mice were crossed with S6k1-/- mice; this almost completely normalized kidney size and cystic index. Surprisingly, the number of BrdU+ and pH3+ cells was not reduced. In contrast, the angle of cell division, which was increased in the Tsc1 fl/fl kidneys, was reduced to baseline by the S6K1-/- cross. This was also seen in an IMCD3 sphere model, in which S6K1 deletion rescued polarization defects and spindle misalignment caused by Tsc1 deletion.

Afadin was identified through an innovative, robust, and well validated screen for S6K targets. Afadin phosphorylation is increased in Tsc1 KO cells and kidneys and decreased in Tsc1/S6K KO cells and kidneys. An alanine mutant of the phosphorylation site was more effective than wild-type in rescuing sphere formation in Tsc1/Afadin KO cells. Finally, in wild-type cells, Afadin localized to the cell boundary/AJ.

The strengths of this work include the identification of a novel S6K substrate that may regulate cell polarization in the kidney. A minor concern is that the term “overgrowth” or “hypergrowth” which are used in the abstract and discussion to describe the kidney phenotype in the double knock out would imply that the kidneys are larger, when in fact they are the same size, roughly, as wild-type. As noted below, how is this possible? Is there a compensatory higher rate of apoptosis?


Specific comments:
1. The differences in localization of Afadin in the WT vs Tsc1KO in Fig 8C are tough to visualize. There seems to be considerable heterogeneity in terms of staining, and it is difficult to conclude based on these images that staining on the Tsc1KO is “more abundant and diffused throughout the whole apical domain” as stated in the text. The Afadin staining in the double KO (top row) looks very different from the WT.

2. Figure 8D: what is the definition of “close” to a single pole? How this was defined and how it was quantitated does not seem to be specified.

3. Page 20: in human trials of TSC, rapalogs decrease the size of angiomyolipomas but to this Reviewer’s knowledge have not been shown to “clearly improve kidney function.” The statement that the doses used did not adequately inhibit mTORC1 is difficult to understand, since lower doses of Rapalogs were used in TSC trials vs ADPKD trials, and clearly in TSC the doses were effective in the kidneys.

4. In the abstract on page 20, the authors state that the hyperproliferation leads to a “harmonious overgrowth” or “hyper-growth” but in fact, despite an increased rate of BrdU labeling, there is no actual overgrowth of the kidney. It would be better to choose a different term than overgrowth or hypergrowth in this situation.

5. What do the authors postulate is actually happening here? How is there increased proliferation, with no organ enlargement? Is there a similarly high rate of apoptosis?

6. Can the authors use human TSC specimens to determine whether Afadin is an S6k substrate in the human disease, and/or that it exhibits mislocalization in cystic epithelia in the mouse or ideally in the human disease? It is recognized that this may not be feasible.

Elizabeth Henske

**Author rebuttal, first version:**

**We thank the three reviewers for their comments and we appreciate they found broad interests in our work. In this revised version, we provide new functional data addressing the role of S6K1 in the pathogenesis of TSC cystic lesions. Briefly, we address three main points. First, we provide further analysis of the kidney development and cell number in S6K1 mutants. Second, we strengthen the connection between Afadin phosphorylation and misoriented cell division by including new data on *in vivo* Afadin phospho-mimetic mutants. Third, we characterized a thorough panel of apico-basal and cell-cell junction markers, thus revealing the peculiar mislocalization of the cadherin-catenin system which accompanies the defect in TSC1-deficient sphere formation. Finally, we performed a whole panel of controls to interpret the cell biology and phospho-proteomic data, as requested by the reviewers. There is a large amount of new data that are included in the 9 figures, and 9 supplementary figures of this revised manuscript.**

**Below is our response to the reviewer’s comments.**

***Reviewer #1:***

*1) Fig 2. Was the spindle angles measured in all tubules? Did they mainly examine the cortex? This is not stated in the methods section. This is important because Ksp-cre recombines in only ~5-10% of proximal tubules, and some of these abnormal measurements could be due to non-cell autonomous defects. Please clarify and address this point.*

**To clarify this important point, in the previous version we quantified the angle of cell division in AQP2-positive epithelial tubules, both in the cortex and in the medulla. AQP2 is a marker of the collecting ducts and the distal tubules. Consistent with previous literature (Igarashi et al., 1999), using a confetti reporter for Cre expression, we detect Cre-mediated recombination both in the cortex and in the medulla (Figure for reviewer R1A and R1B below). In this revised version, we also quantified WGA -positive and - negative tubules in the whole kidney. WGA is a marker of proximal tubules. Again, as shown below (Figure for reviewer R2A and B), both WGA -positive and -negative tubules display a misoriented cell division in TSC1 mutant kidneys. Thus, our data are consistent in both cortex and medulla, in different segments of the tubules. Since the second reviewer suggested to increase the number of mitotic events measured, we decided to include all the data in the new Figure 2G. It is possible that non-cell autonomous effects also contribute to cyst formation, once the kidney organization is considerably altered. However, our in vivo data at pre-cystic stage as well as the in vitro**

**data on sphere formation indicate an important cell-autonomous component triggering misoriented cell division. The observed alterations in cell-cell adhesion could also represent a way to expand the phenotype to neighboring cells.**

**A	B**

Figure R1. High magnification of cortex (A) and medulla (B) of a pre-cystic *R26R-Confetti; Ksp-Cre; Tsc1 f/f* animal. The multi-color confetti reporter is here shown in grey-scale, to appreciate the spreading of Cre-positive cells independently on the reporter channel.

**A	B**

Figure R2. Quantification of the mitotic division orientation at post-natal day 20 of the different genotypes indicated. Data from AQP2-positive and WGA-positive and negative (A) or AQP2-positive and WGA-negative (B) tubules were pooled and the distribution compared.

2) *Fig 4. A cyst that does not have a clear central lumen (i.e. clear demarcation of apical and basal) cannot be used to assess spindle angle, as they do for the TSC knockouts in Figure 4. This is because there is no true frame of reference.*

**Both reviewers #1 and #2 are correct that apical and basal markers are important to adequately measure the center of the spheroid. In this revised version, we provided more images and we included a variety of markers. From this analysis, it is clear that TSC1 deficient spheres correctly position a variety of apical and basal markers: Par3, aPKC, ZO-1 and Ezrin apical markers and Integrin-β-1 basal marker (Fig. 5A, 5B, new Suppl. Fig. S6A, S6B and S6C respectively). In suppl. Figure S5C, we also provide the images of the 3D reconstitution and of the orthogonal projections after phalloidin and Par3 staining in the spheroids of the three genotypes. Taking into account all these data, it is straightforward to define the center of the spheroids and provide evidence that TSC1 knockout spheres do have a clear central lumen. As suggested by the reviewer #2, we also measured an additional parameter to provide quantitative data on sphere formation: the ratio of the lumen diameter to the number of cells surrounding the lumen (Suppl. Figure S5A). The data from 100 sphere measurements are consistent with the decrease in lumen size of TSC1 deficient spheres.**

3) *The authors show that the TSC1 KO cells have more Afadin and more phospho-Afadin than wild type and TSC/S6K1 KO cells. Their data demonstrates that S6K1 phosphorylates Afadin, and they suggest that the phosphorylation promotes Afadin protein stability. However, this can (and should) be tested directly in vitro to support or refute this claim.*

**In this revised version, we also measured the mRNA levels for afadin and found that TSC1 deficient cells have increased mRNA levels (new suppl. Fig. S8C). Therefore, the increase in afadin expression in TSC1 deficient cells likely reflects a complex regulation, combining effects at different levels, on both transcription and protein stability. To dissect the functional role of Afadin phosphorylation, we favored approaches using the phospho-mimetic mutants (see below).**

4) *Also, if it is increased phospho-Afadin that promotes misoriented OCD, then overexpression of a phospho-mimetic at this site in wild type would be expected to have the same effect. This is a critical experiment that should be done.*

**This is an important point that led us to the experiment in the new Figure 8C. Importantly, the expression of the acidic phospho-mutant was sufficient to alter sphere formation in wild type cells. In addition, in 2D cultures at confluency the acidic mutant had clearly an impact on the shape of cell-cell junctions (new suppl. Fig. S9D).**

5) *In Fig 8, they report that afadin staining is increased at the apical/lumenal side in TSC mutants compared with control. This experiment needs to be done in conjunction with an apical marker and with a junctional marker (e.g. ZO1) because some of the afadin that appears apical can in fact be apical junction due to the plane being visualized. Also, is the phospho-Afadin at the apical site?*

**We thank the reviewer for raising this point. In this revised version, a large panel of antibodies against apico-basal and junctional markers was used. As shown in the new suppl. Fig. S5, both the Par3, aPKC, and Ezrin apical markers and basal marker Integrin-β-1 basal are correctly localized in TSC1 mutants (Fig. 5A, 5B, new Suppl. Fig. S6B and S6C respectively). The junctional marker ZO-1 is also correctly positioned relative to afadin (new Fig. 9C and new suppl. Fig. S6A and S10A). It is possible that the apparent afadin and ZO-1 enrichment at the apical domain may also be due to the shrinkage of the lumen and reduced surface of the apical domain in the TSC1 mutants. However, in this revised version we also included immunofluorescence analysis in kidney sections at the pre-cystic stage in vivo. As shown in the new Fig. 9D, Afadin expression tends to decorate the whole apical domain in TSC1 mutant kidneys while it is more restricted to the apico-lateral cell-cell junctions in wild type and TSC/S6K1 deficient cells. What is clearly altered in the TSC1 deficient spheres are the E-cadherin, α- and β-catenin and Scribble staining (new Fig. 9A, 9C, suppl. Fig. S6C, S10, S11A and S11B), pointing to major defects in the regulation of the cadherin adhesion system (see below). Unfortunately, the phospho-specific antibody against afadin works well in immunoblot analysis but not in immunofluorescence studies, thus precluding the analysis of phospho-afadin localization. We revealed this lack of specificity in the immunofluorescent detection of phospho-afadin using our afadin knockout cells.**

6) *In Fig 8 the authors report that the junctions are mispositioned. However, if there is no normally-located lumen, it is not clear that the junctions are the issue. Also, Ecadherin appears globally reduced, even at the later borders of cells, based on the IF. The location of Ecadherin*

*at these sites it not expected to be altered by changes in afadin, which is normally restricted to the apical-junctional complex. Please address.*

**As reported in the point #2 above, in this revised version we provided evidence that TSC1 deficient spheres do form a lumen with correct apical and basal organization. A series of basolateral markers showed correct localization in TSC1 deficient spheres (new Suppl. Fig. S6 ). However, not only E-cadherin but also α- and β-catenin and Scribble are abnormally present in the apical domain (new Fig. 9A, 9C, suppl. Fig. S6C, S10, S11A and S11B). This is a striking phenotype that may have a major role on oriented cell division, which is known to rely on the cues from the cell-cell adhesion systems. It is likely that afadin phosphorylation may be involved in the alteration of cadherin localization, as the acidic mutant shows an effect.**

7) *In the discussion they report that “Centrosome misposition in mitotic cells underlies the defect in oriented cell division”. The data suggests the two are correlated in that both are abnormal, but the data does not show any causality. Thus, the conclusions are not supported by the results.*

**The reviewer is correct and the sentence was modified to indicate the correlation: “Centrosome misposition in mitotic cells accompanies the defect in oriented cell division”**

8) *Loss of Afadin has been shown to cause misoriented cell division in vitro and in vivo in renal tubules. The authors should cite this early in the manuscript, as it is central to the theme and not mentioned until nearly the end of the discussion. How do the authors explain that loss of Afadin causes misoriented cell division in the other study and that increased expression of phospho-Afadin causes causes loss of oriented cell division in their study?*

**In this revised version, we further discussed the data from the Afadin knock­out study. The complete loss of afadin alters sphere formation, but does not exactly phenocopies the TSC1 deficient cells. Afadin deficiency also leads to multi-lumen formation, which is not observed in our TSC1 deficient spheres. Our hypothesis is that afadin phosphorylation somehow impairs the control of the cadherin system, thus leading to misoriented cell division due to altered cell-cell adhesion cues.**

*Minor	point*

*1) Fig S1: please plot data in columns as individual values rather than mean/median, as you do in Fig 1.*

**We modified the graph (new suppl. Fig. S3B).**

***Reviewer #2:***
*Major:*

1. *Figure 1A. It appears that there are small cysts in the Ksp-Cre; Tsc1 f/f; S6k1 -/- kidneys. A Supplemental Figure should be made with both medium and high power images from all genotypes, multiple panels from different kidneys, to give the reader a full appreciation of histology and pathology in these mouse kidneys.*

**In the new suppl. Fig. S1, we provided more images at different magnification of the kidney sections. The TSC1/S6K1 knockout kidneys do display some small cysts, but their cystic index is eight time smaller than the TSC1 knockout (Fig. 1C).**

2. *What happens to the increased number of cells generated by division in the Tsc1KO S6K1 KO kidneys. Is there a net gain in the number of cells without expansion of the kidney size? Seems a bit odd. Can you quantify total cell counts in those kidneys? This may connect with requested high power views of the kidneys in 1, above. Note that Figure E and G left panels, controls, are not adequate, as they do not show normal renal tubules, 1G left is substandard, a washed out image as well.*

**We thank the reviewer for this important point. The increased kidney weight of the TSC1 deficient mice is due to increased proliferation and fluid retention. The TSC1/S6K1 mutants do have an expansion of the kidney size as compared to control and S6K1 knockout mice (Fig. 1B). Their decreased kidney weight as compared to TSC1 mutants is mainly due to the lack of polycystosis and fluid retention. In this revised version, we provided additional evidence on the growth of TSC1/S6K1 mutants. As shown in the new suppl. Fig S2A and S2B, the cortex to medulla ratio is reduced in the TSC1/S6K1 mutants as compared to the wild type. These data are consistent with this genotype having an elongation of the tubules of the nephron, which are predominantly**

**in the medulla while the glomeruli are in the cortex. Finally, we improved the histology images as requested by the reviewer.**

3. *Figure 2A should include a Tsc1 blot.*

**Unfortunately, the quality of the immunoblot analysis from kidney extracts using an anti-TSC1 antobody is not adequate. In the new Fig. 2A, the levels of TSC1 expression were assessed by QRTPCR analysis, indicating the extent of TSC1 deletion in both TSC1 and TSC1/S6K1 mutant genotypes.**

4. *For both the ksp-cre and CAGG-cre models, it seems that not all cells are dividing at any time. Why not?*

**In these experiments, the pulse of BrdU was 2 hours prior to sacrifice. Therefore, only a fraction of cells are in S-phase during this time interval.**

5. *Figure 2E and F. This needs to be demonstrated better. Add S figure to show many of these cells captured in mitosis by this technique. In addition, n=30 mitotic events is not enough, please do at least 100 each. Why are so few mitoses at an angle near 0° in the control mice?*

**It is to note that finding mitosis in anaphase, in 20-day-old mice, in AQP2 positive tubules is an extremely rare event (a maximum of 1 in a 0.4x0.4x1 mm section in the controls, a maximum of 4 in *Ksp-Cre; Tsc1 f/f* and *Ksp-Cre; Tsc1 f/f; S6k1 -/-*). To answer both reviewers #1 and #2 (see point #1 of reviewer #1), in this revised version we measured the angle of cell division in AQP2-positive and -negative cells, as well as in WGA-positive and -negative cells. The distribution of cell division angles is similar (see Fig. for reviewer R2 above). To increase the number of events, we therefore included all data from the different kidney regions and arrived to a number of events of 41 for the *Tsc1 f/f* , 80 for the *Ksp-Cre; Tsc1 f/f* and 49 for the *Ksp-Cre; Tsc1 f/f; S6k1 -/-* mice (new Fig. 2G). Finally, the distribution of mitosis in control mice are consistent with other studies (Kunimoto et al., 2017; Saburi et al., 2008) which have a median angle of 14-20°, comparable with ours, that is 20°. To explain why we detect so few mitoses at an angle near 0° in the control mice, we can refer to the probability to detect a dot on the surface of a sphere, being the sphere the orbit defining the distribution of probability to measure**

**an angle of mitosis between 0 and 90° relatively to the tubular lumen. In this scenario, close to the poles of the sphere (0-10° angle) the surface is smaller than the area between 0-20°, therefore shrinking the probability to find a very small angle in this system. Finally, we could measure a lower amount of mitosis in the controls due to the fact that, at post-natal day 20, the large majority of renal cells stop proliferating.**

6. ***‘The phosphorylation of ribosomal protein S6 (RPS6) was not completely inhibited in S6K1*** *de****ficient kidneys, owing to the presence of S6K2’ Is there evidence of this from previous*** *studies, or in work the authors can show? Figure 2A shows complete absence of S6K in the S6K1-/- kidneys, suggesting there is no expression of S6K2.*

**In previous studies, we demonstrated that S6K2 can effectively phosphorylate rpS6 in S6K1 deficient tissues (Pende et al., 2004). Actually, in a number of tissues the rpS6 phosphorylation is more reduced in S6K2 knockout cells than in S6K1 knockout cells. The antibody that we used in Fig. 2B is specific for S6K1 and does not cross-react with S6K2. Finally, in S6K1-S6K2 deficient kidneys, rpS6 phosphorylation is abrogated, demonstrating the involvement of S6K2 (see below Figure for reviewer R3).**

**Figure R3. Western blots of mouse whole-kidney extract at 3-months of age of mice of the indicated genotypes. Mice are randomly fed. Antibodies against pS6K^T389^ and pRPS6^s240-244^ are used. Actin is shown as loading control.**

3. *My eye sees that pAKT levels are pretty similar across Figure 2A blot, in contrast to the statement in the text.*

**The reviewer is correct. There was a tendency that did not reach statistical significance but it’s not worth mentioning this point in the text and we removed the statement.**

8. *Figure 4C top does not look like the other images of Tsc1 KO/S6k1 KO cells. The cells are bunched up and not forming a nice spheroid. Please generate some quantitative or semi-quantitative measure of spheroid formation, e.g. lumen to cell # ratio, and measure this for large numbers of spheroids, to provide quantitative data for each genotype.*

**We thank the reviewer for this important point that was also raised by reviewer #1. See point # 2 of reviewer #1.**

9. *Figure 6 A label in the figure says AKT volcano plot while the legend says AGC kinase-motif. This figure goes out of focus when seen at high enough mag to read the labels. Please add in the image if red means up in which genetic mix vs. which other one. The figure legend is also not clear on this point; green means up in Tsc1 KO vs. Tsc1 KO/S6K1 KO?*

**We modified the figure and legend according to the reviewer comments.**

0. *Was there only one phosphorylation site for afadin identified in the mass spec analysis? Multi-site phosphorylation is common.*

**This is the only site with an AGC kinase consensus site. There was an additional site that was decreased in S6K1 mutant cells after IMAC enrichment (Ser1719) but it is unlikely to be directly phosphorylated by S6K1 because of the consensus sequence.**

1. *The images in Figure 8C are crucial, and are not well-explained. What are all the small squares with arrows and some colors? It seems that the columns of figures are different examples for each genotype, but this is not clear. The legend does not explain well either. Why is there so much variability, some cells seem to express no E-cadherin at all, while in others it is abundant?*

**This is an important point that led us to further analysis of the adhesion system, using additional antibodies including α- and β-catenin and Scribble. As shown in the new Fig. 9A and 9C and in new suppl. Fig. S6C, S10, S11A and S11B there is a striking mislocalization of these proteins in TSC1 deficient spheres. This was also confirmed in vivo (new Fig. 9D and 9E). In addition, we improved the explanation of Fig. 9A in the legend.**

*12. Please do IHC on the mouse kidneys, all 3 genotypes, to show afadin and p-afadin localization in both resting and mitotic cells.*

**We thank the reviewer for raising this point that improved our study. In the new Figure 9D, we performed Afadin immunofluorescence analysis in kidney sections. Unfortunately, the phospho-specific antibody is not suitable for immunofluorescence analysis, as shown using afadin knockout cells as negative controls. However, the antibody against total afadin is very specific and confirmed in vivo the alteration in afadin localization that was observed in cultured spheres. In TSC1 deficient kidneys afadin is not restricted at the cell-cell junction but tends to decorate the whole apical domain.**

*Minor comments:*

1. *‘In addition, TSC patients display increased risk to develop polycystic kidney disease, which may represent an important morbidity and mortality factor.’ This is an overstatement. 2-3% of TSC patients develop severe PKD due to concurrent genomic mutation in TSC2 and PKD1. Other TSC patients have kidney cysts, but they are few in number, and not a source of morbidity or renal dysfunction.*

**We agree with the reviewer on this point and clarified the text.**

2. *‘mediate cyst formation are elusive.’ Prefer unknown rather than elusive. No one has looked before, does not mean they are elusive.*

**We modified the text.**

*3. ‘phosphorylation of other mTORC1 substrates may be relatively spared’ There is quite good*

*evidence from past studies from the Sabatini lab that sensitivity of mTORC1 to rapalog inhibition varies a great deal among substrates with S6K1 being the most sensitive.*

**We completely agree, these papers are cited and very relevant to our work on S6K deletion.**

4. *Further suggestive evidence that S6Ks are driving cyst formation is the sensitivity of cyst formation in various genetic models to rapalog therapy, given the sensitivity of S6K phosphorylation to rapalogs, and relative resistance of other substrates.*

**We thank the reviewer for raising this point which was included in the discussion.**

5. *‘plasmatic urea levels’ should be serum urea levels; at least that is the usual blood derivative used for urea measurement.*

***We modified the figure and text accordingly.***

6. *‘a net amelioration of’ replace with ‘improvement in’* **We modified the text.**
7. *‘up-regulates the rate of cell proliferation, though the tubular cells are protected against tubular enlargement.’ Prefer ‘increases cell proliferation but cyst formation does not occur.’*

**We modified the text.**

8. *‘a major determinant of cyst formation’ prefer ‘required for robust cyst formation’.*

**We modified the text.**

9. *Mouse gene names (re: mIMCD3 cells) should be italicized with first letter only in caps. E.g. Tsc1 for mouse, not TSC1.*

**We modified the text.**

2. *‘with an average of around 16 cells corresponding to 5 doubling times’ 2**4=16, hence 16 cells corresponds to 4 doubling events or sets of cell division.*

**We modified the text.**

3. *‘In human clinical trials, rapamycin derivatives clearly improved kidney function in TSC patients 69, while the results against ADPKD were moderate or negative 70, 71, 72.’ This statement is misleading. The benefit of rapamycin in TSC kidneys is mainly due to reduction in size of angiomyolipoma, leading to improved kidney function. Angiomyolipomas do not occur in ordinary ADPKD.*

**Both reviewers #2 and #3 are correct. We introduced these points of caution in the discussion.**

4. *It’s hard to appreciate the magnitude of the difference in the red vs. other color dots in Fig 3CD. Please figure out a presentation method that makes this easier to see.*

**We modified the dots.**

***Reviewer #3:***

*Specific comments:*

*1. The differences in localization of Afadin in the WT vs Tsc1KO in Fig 8C are tough to visualize. There seems to be considerable heterogeneity in terms of staining, and it is difficult to conclude based on these images that staining on the Tsc1KO is “more abundant and diffused throughout the whole apical domain” as stated in the text. The Afadin staining in the double KO (top row) looks very different from the WT.*

**We thank the reviewer for this point which was also raised by the other reviewers (see above). In this revised version, we included the following analysis to strengthen our conclusion (new Figure 9 and suppl. Fig. S6, S10 and S11): i) additional components of the cadherin system, including catenin and Scribble; ii) detection of afadin in kidney section. Our data demonstrate a striking alteration of the localization of the cadherin cell-cell adhesion system, which becomes localized at the apical domain in the TSC1 deficient cells.**

2. *Figure 8D: what is the definition of “close” to a single pole? How this was defined and how it was quantitated does not seem to be specified.*

**By saying that the afadin spot is close to a single pole we meant that, in the Tsc1 KO spheres, that a single Afadin spot (yellow arrows in insets of Fig. 9A and suppl. Fig. S10A) is found on only one side of the spindle-equator of metaphase cells (white dotted line in the same insets). This is opposite to what we observe in the other two genotypes, where 2 distinct Afadin pooles are found at the two sides of the spindle-equator. We clarified this issue in the text.**

3. *Page 20: in human trials of TSC, rapalogs decrease the size of angiomyolipomas but to this Reviewer’s knowledge have not been shown to “clearly improve kidney function.” The statement that the doses used did not adequately inhibit mTORC1 is difficult to understand, since lower doses of Rapalogs were used in TSC trials vs ADPKD trials, and clearly in TSC the doses were effective in the kidneys.*

**Both reviewers #2 and #3 are correct. We introduced these points of caution in the discussion.**

3. *In the abstract on page 20, the authors state that the hyperproliferation leads to a “harmonious overgrowth” or “hyper-growth” but in fact, despite an increased rate of BrdU labeling, there is no actual overgrowth of the kidney. It would be better to choose a different*

*term	than	overgrowth	or	hypergrowth	in	this	situation.*

4. *What do the authors postulate is actually happening here? How is there increased proliferation, with no organ enlargement? Is there a similarly high rate of apoptosis?*

**We thank the reviewer for these two points which were also raised by the reviewer #2 (see point #2 reviewer #2).**

*6. Can the authors use human TSC specimens to determine whether Afadin is an S6k substrate in the human disease, and/or that it exhibits mislocalization in cystic epithelia in the mouse or ideally in the human disease? It is recognized that this may not be feasible.*

**This is an important point that led us to addressing afadin phosphorylation by immunoblot analysis in human biopsies from TSC patients. We used this technique because the antibody does not work in ummunohistochemistry. The data in Fig. 7E and 7F show increased Afadin phosphorylation in TSC patients compared to non-TSC patients.**

**Reviewer comments, second version:**

Reviewers' comments:

Reviewer #1 (Remarks to the Author):

Overall the manuscript is somewhat improved, however, some of the additional experiments are discrepant with their original hypothesis. There are several examples in which the conclusions are not supported by certain experiments, but this is ignored. Thus, while the data is novel and interesting, I remain concerned with its veracity. Specific comments are below.

1) In Figure 2, the proximal tubules of mutant Tsc mice have abnormal oriented cell division, same as the collecting ducts. Since only 5-10% of proximal tubules will have recombined (and thus will lack Tsc), this strongly points to a non-cell autonomous defect, perhaps due to distorted architecture and/or cystogenesis. If it is non-cell autonomous, this result is not consistent with their hypothesis. To address this in part, they would need to repeat the experiments with a reporter allele that indicates which cells have undergone recombination. They would also need to show the results in precystic tubules. In the text, they state that they did these experiments, but it is not shown. It would also be desirable to really indicate the dividing cells are part of the tubule and not the underlying stromal cells (some images of this would suffice). Finally, when presenting the results, the proximal tubule and collecting duct data should be shown separately, not combined.
2) As noted in the prior review, Tsc1 mutants have increased phospho-Afadin AND total Afadin. The authors have addressed this point in part, showing that Tsc1 KO cells have increased Afadin mRNA. Thus, they cannot state that phosphorylation may contribute to Afadin stability. In fact, their data indicate otherwise (Fig 8b).
3) To address whether phosphorylation of Afadin is responsible for the mis-oriented cell division, they perform additional experiments (e.g. Fig 8). However, important controls are lacking and the data are not consistent. For example, they show relative rescue of “sphere formation” in the Afadin KO spheroids with empty vector, wild type, and S1795A afadin, however, the experiment lacked comparison to control spheroids, which would indicate the relative degree recue compared to controls, and also lacked testing of the phosphomimetic Afadin. Importantly, the spheroids were rescued equally with wild type and S1795A afadin, which does not support their hypothesis. They also don’t show OCD measurements, just “sphere formation”. In the TSC1/Afadin DKO experiments, they do show that the S1795A rescues better than wild type afadin, although the degree of rescue relative to controls is again not shown. Ideally, they could have performed an experiment in the Afadin KO lines with the S1795A and S1795E, but they only use phosphomimetic S1795E Afadin in experiments with wild type cells. In that experiment, the levels of expression of phosphomimetic Afadin are not shown and there appears to be mislocalization of afadin (with increased cytosolic afadin) in these mutants. All in all, I think that the data are not conclusive and it would be misleading to the field to publish it with these questions lingering.
4) They state that the phosphorylation of afadin may be involved in the mislocalization of cadherin. The data does not suggest if this is true or false.
5) Since both Afadin and phospho-Afadin are increased in the Tsc1 mutants, it is possible that changes in the total level of Afadin could explain part of the phenotype. Please address.
6) The new experiments showing mislocalization of cadherins in Tsc1 mutants is novel and appears robust and convincing.


Reviewer #2 (Remarks to the Author):

The response to the prior review is comprehensive, and overall well-done. I have no major issues. I raise the following minor issues.
Minor issues:
1. ‘Plasmatic urea’ is still present in Figure 1D and used in the text.
2. Figure S2AB. Why would medullary expansion be greater in the Ksp-Cre+ Tsc1ffS6k1— mice than in the Ksp-Cre+Tsc1ff mice?
3. Figure 2G. Mean is a poor choice for placement of the main bar, since the distribution is not normal. Place the main bar at the median, and use box to show 80 or 90% of dots, will be more informative. Otherwise, the statement ‘the great majority of mitotic events the angle of cell division relative to the lumen was less than 20°’ is not supported well by the figure.
4. The afadin label in Figure 6A is not done well. I can’t tell which dot it is associated with, please improve. Please add afadin label to 6B as well.


Reviewer #3 (Remarks to the Author):

all of my concerns have been thoroughly and thoughtfully addressed

**Author rebuttal, second version:**

**Thank you for your continued interest in our study. All three reviewers recognize that we have thoroughly demonstrated these novel issues: 1) the role of S6K1 in mTOR-driven cystogenesis; 2) the *in vitro* and *in vivo* evidence for a specific, yet coordinated control of oriented cell division (OCD) and proliferation rate; 3) a broad S6K1 phospho-proteome, revealing for the first time a class of substrates involved in cell-cell adhesion and actin binding; 4) Afadin as a novel S6K1 substrate participating in the control of OCD; 5) the S6K1-dependent regulation of the cadherin adhesion system.**

**We have analysed these questions in depth, by using both *in vivo* and *in vitro* systems, such as mouse models, human biopsies, and a variety of edited kidney cell lines grown in 3D spheroids.**

**The first reviewer argues that we should be more cautious on the cell autonomous control of OCD and on the molecular mechanism(s) affected by Afadin phosphorylation. We largely agree with him/her. In this revised version, we address some of his/her concerns and modify several of our statements regarding these two issues. Please see below our answers to the specific points.**

**Reviewer #1 (Remarks to the Author):**

Overall the manuscript is somewhat improved, however, some of the additional experiments are discrepant with their original hypothesis. There are several examples in which the conclusions are not supported by certain experiments, but this is ignored. Thus, while the data is novel and interesting, I remain concerned with its veracity. Specific comments are below.

1) In Figure 2, the proximal tubules of mutant Tsc mice have abnormal oriented cell division, same as the collecting ducts. Since only 5-10% of proximal tubules will have recombined (and thus will lack Tsc), this strongly points to a non-cell autonomous defect, perhaps due to distorted architecture and/or cystogenesis. If it is non-cell autonomous, this result is not consistent with their hypothesis. To address this in part, they would need to repeat the experiments with a reporter allele that indicates which cells have undergone recombination. They would also need to show the results in precystic tubules. In the text, they state that they did these experiments, but it is not shown. It would also be desirable to really indicate the dividing cells are part of the tubule and not the underlying stromal cells (some images of this would suffice). Finally, when presenting the results, the proximal tubule and collecting duct data should be shown separately, not combined.

**As shown in the new Fig. S1, a confetti reporter shows that there is significant recombination in both the cortex and medulla. In this revised version, we show the measurements of cell division angles separately for the medulla (Fig. 2G) and cortex (Supplementary Fig. S5B), as suggested by the reviewer. In addition, we provide immunofluorescence pictures before 3D reconstruction, showing that cell division is measured in epithelial cells lining the tubule and not in underlying stromal cells (Supplementary Fig.**

**S5A). Measurements of OCD were performed on original pictures and not on 3D reconstructed images, in order to distinguish the tubular cells from the stromal ones. All these experiments were performed at P20, therefore at a precystic stage.**

**It is very likely that non-cell autonomous effects also participate in tubule distortion. These could be humoral factors, disturbed urinary flow or altered cellular adhesion between mutant cells and neighbouring wild type cells. There was already some evidence that Tsc1 deletion in the kidney can lead to non-cell autonomous activation of the mTORC1 pathway in epithelial cells (Zhou et al, PMID 19692352). Therefore, mTORC1 is likely to alter OCD in both Tsc1 mutant and neighbouring wild type cells, consistent with the general efficacy of rapamycin to blunt polycystic disease. We formulate these possibilities in the revised text, highlighted in yellow. However, in this study, we focused on cell autonomous effects by generating a whole battery of IMCD3 genetically edited cells for *in vitro* studies.**

2) As noted in the prior review, Tsc1 mutants have increased phospho-Afadin AND total Afadin. The authors have addressed this point in part, showing that Tsc1 KO cells have increased Afadin mRNA. Thus, they cannot state that phosphorylation may contribute to Afadin stability. In fact, their data indicate otherwise (Fig 8b).

**We sincerely apologize and thank the reviewer for pointing out the inconsistency of Supplementary Fig. S8B of the previous revised version (now Supplementary Fig. S10B). The two genotypes were mislabelled in the previous version. We corrected this error in this revised version and show that Afadin protein stability is increased in the *Tsc1* mutants. We agree, and state in the text, that the precise molecular mechanisms regulated by Afadin phosphorylation requires further investigation. There is a correlation between Afadin phosphorylation and stability, but we did not demonstrate a causal relationship. Afadin phosphorylation could also affect molecular interactions at the actin cortex and adhesion complexes. We have also evidence that phospho-mimetic Afadin mutant has impaired localization at cell-cell junctions (Supplementary Fig. S11E and S11F). This should be clearly addressed in future studies.**

3) To address whether phosphorylation of Afadin is responsible for the mis-oriented cell division, they perform additional experiments (e.g. Fig 8). However, important controls are lacking and the data are not consistent. For example, they show relative rescue of “sphere formation” in the Afadin KO spheroids with empty vector, wild type, and S1795A afadin, however, the experiment lacked comparison to control spheroids, which would indicate the relative degree recue compared to controls, and also lacked testing of the phosphomimetic Afadin. Importantly, the spheroids were rescued equally with wild type and S1795A afadin, which does not support their hypothesis. They also don’t show OCD measurements, just “sphere formation”. In the TSC1/Afadin DKO experiments, they do show that the S1795A rescues better than wild type afadin, although the degree of rescue relative to controls is again not shown. Ideally, they could have performed an experiment in the Afadin KO lines with the S1795A and S1795E, but they only use phosphomimetic S1795E Afadin in experiments with

wild type cells. In that experiment, the levels of expression of phosphomimetic Afadin are not shown and there appears to be mislocalization of afadin (with increased cytosolic afadin) in these mutants. All in all, I think that the data are not conclusive and it would be misleading to the field to publish it with these questions lingering.

**We would like to stress that in our study we produced four novel IMCD3 cell lines by genome editing: *Tsc1*, *Tsc1/S6k1*, *Afadin*, and *Tsc1/Afadin* mutants as compared to wild type cells. We isolated two edited clones for each mutant and performed spheroid assays in biological triplicates. In rescue experiments, we stably transduced cells with lentiviral vectors expressing wild type, phospho-mutant and phospho-mimetic *Afadin* cDNAs. To assay a manageable number of dishes, we performed the rescue experiment in the optimal genetic background to see an effect of the phosphorylation mutants: i.e. for the phospho-mimetic mutant, we used the wild type background (low phosphorylation of endogenous Afadin), while for the phospho-mutant we used the *Tsc1/Afadin* background. For this screening, we scored for sphere formation as we have previously shown it correlates well with OCD (Fig. 4C). In this revised version, we express all the graphs as % of polarized spheres, as suggested by the reviewer (Fig. 8B and 8D, supplementary Fig. S11D). In the previous version, we opted to show fold changes because the two *Afadin* KO clones and the two *Tsc1/Afadin* clones had a slight difference in the basal spheroid %. However, the results are still significant if we present them as absolute values (Fig. 8B and supplementary Fig. S11D). We agree with the reviewer that it is easier to appreciate now the extent of rescue, as compared to the other genotypes. Clearly, Afadin phosphorylation concurs together with additional targets in the alterations of sphere formation. We stated this issue in the text, highlighted in yellow.**

**The reviewer is correct that we also need to show the expression levels for the phospho-mimetic mutant. In the new Supplementary Fig. S11B we show that the E mutant levels are comparable to the others. We also changed to a more representative image of the immunofluorescence localization (Supplementary Fig. S11E). When we exogenously increase the expression of all forms of Afadin, we detect more cytosolic staining as compared to endogenous (see Supplementary Fig. S11C, where both the WT form and the A-mutant have comparable cytosolic expression). However, we find less junctional E-mutant as compared to the A-mutant (Supplementary Fig. S11E and S11F). In conclusion, the expression of the E- and A-mutants are comparable to the wild type Flag-Afadin, though only the E-mutant is less junctional and decreases the ability to form spheroids. This is an argument that Afadin phosphorylation specifically concurs to the observed effects on sphere formation. It is possible that Afadin overexpression in the *Tsc1*-mutant background also plays a role, though we did not address it in this study.**

4) They state that the phosphorylation of afadin may be involved in the mislocalization of cadherin. The data does not suggest if this is true or false

**The reviewer is correct. We now stress in the text that this is a correlation and not a causal relationship, highlighted in yellow. We made this hypothesis based on the literature showing Afadin interaction with the Cadherin adhesion systems.**

5) Since both Afadin and phospho-Afadin are increased in the Tsc1 mutants, it is possible that changes in the total level of Afadin could explain part of the phenotype. Please address.

**See above, last sentence in the answer to point #3**

6) The new experiments showing mislocalization of cadherins in *Tsc1*-mutants is novel and appears robust and convincing.

**We thank the reviewer for recognizing this novelty.**

**Reviewer #2 (Remarks to the Author):**

The response to the prior review is comprehensive, and overall well-done. I have no

major issues. I raise the following minor issues.

Minor issues:

1. ‘Plasmatic urea’ is still present in Figure 1D and used in the text.

**We assayed Plasmatic Urea. In many references, plasma urea is used as much as serum. See for instance the recommendations from the Clinical Biochemistry Association, UK**

[**http://www.acb.org.uk/Nat%20Lab%20Med%20Hbk/Urea.pdf**](http://www.acb.org.uk/Nat%20Lab%20Med%20Hbk/Urea.pdf)

2. Figure S2AB. Why would medullary expansion be greater in the Ksp-Cre+ Tsc1ffS6k1— mice than in the Ksp-Cre+Tsc1ff mice?

**We thank the reviewer for raising this point. In the previous version, we expressed the results as a cortex/medulla ratio, focusing on the decrease of the ratio for the *Ksp-Cre +/-*; *Tsc1*^f/f^; *S6k1 -/-* kidneys as compared to the controls *Tsc1 f/f*. (Supplementary Fig. S3B). This result confirms the sustained proliferation in the *Ksp-Cre +/-*; *Tsc1*^f/f^; *S6k1 -/-* animals and indirectly demonstrates that the proliferating cells concur to the lengthening of the tubules (reason why the medulla, almost exclusively occupied by tubules, increases in size). In this revised version, we clarify this issue by adding the absolute values for cortex and medulla thickness (Supplementary Fig. S3C). The medullar thickness is comparable in the two *Tsc1*-mutant genotypes, due to the fact that in both there is a proliferative phenotype of the tubular cells.**

3. Figure 2G. Mean is a poor choice for placement of the main bar, since the distribution is not normal. Place the main bar at the median, and use box to show 80 or 90% of dots, will be more informative. Otherwise, the statement ‘the great majority of mitotic events the angle of cell division relative to the lumen was less than 20°’ is not supported well by the figure.

**We thank the reviewer for ameliorating the quality of our figure, which has been modified in this revised version. We now show the results as box and whiskers plot also maintaining the single data dots. The graphs therefore shows: min, max, median, first quartile, third quartile and interquartile range.**

4. The afadin label in Figure 6A is not done well. I can’t tell which dot it is associated with, please improve. Please add afadin label to 6B as well.

**We modified the figure and highlight Afadin in yellow in both screens. The Afadin phosphorylation is less evident in the IMAC enrichment, probably because the more specific enrichment in the AGC-motif screen allowed better recovery of that specific peptide and thus more reproducible quantification.**

Reviewer #3 (Remarks to the Author):

all of my concerns have been thoroughly and thoughtfully addressed

**Reviewer comments, third version:**

REVIEWERS' COMMENTS:

Reviewer #1 (Remarks to the Author):

My comments have largely been addressed by rewording and toning down their conclusions/interpretations of the data.

In response to my review, the authors state: “It is very likely that non-cell autonomous effects also participate in tubule distortion” in mutant Tsc mice. This is downplayed—in fact it may be that non-cell autonomous defect are a primary/major reason for tubule distortion based on their in vivo results showing defects in OCD in the cortex, much of which consists of proximal tubules that are minimally recombined in the Ksp-cre Tsc mice. Adding a statement that non-cell autonomous effects could be a major reason for the tubule distortion should suffice.

**Author rebuttal, third version:**

Reviewer #1 (Remarks to the Author):

My comments have largely been addressed by rewording and toning down their conclusions/interpretations of the data.

In response to my review, the authors state: “It is very likely that non-cell autonomous effects also participate in tubule distortion” in mutant Tsc mice. This is downplayed— in fact it may be that non-cell autonomous defect are a primary/major reason for tubule distortion based on their in vivo results showing defects in OCD in the cortex, much of which consists of proximal tubules that are minimally recombined in the Ksp-cre Tsc mice. Adding a statement that non-cell autonomous effects could be a major reason for the tubule distortion should suffice.

**We modified the sentence in the Results section as it follows:**

**It is likely that non-cell autonomous effects could be a major reason of tubule distortion, including humoral factors, disturbed urinary flow or altered cellular adhesion between mutant cells and neighbouring wild type cells.**
